# Grapevine as a Rich Source of Polyphenolic Compounds

**DOI:** 10.3390/molecules25235604

**Published:** 2020-11-28

**Authors:** Iva Šikuten, Petra Štambuk, Željko Andabaka, Ivana Tomaz, Zvjezdana Marković, Domagoj Stupić, Edi Maletić, Jasminka Karoglan Kontić, Darko Preiner

**Affiliations:** 1 Department of Viticulture and Enology, Faculty of Agriculture, University of Zagreb, 10000 Zagreb, Croatia; isikuten@agr.hr (I.Š.); pstambuk@agr.hr (P.Š.); zandabaka@agr.hr (Ž.A.); zmarkovic@agr.hr (Z.M.); dstupic@agr.hr (D.S.); emaletic@agr.hr (E.M.); jkkontic@agr.hr (J.K.K.); dpreiner@agr.hr (D.P.); 2 Centre of Excellence for Biodiversity and Molecular Plant Breeding, Faculty of Agriculture, University of Zagreb, 10000 Zagreb, Croatia

**Keywords:** grapes, secondary metabolites, polyphenolic compounds, phenolic acids, human health

## Abstract

Grapes are rich in primary and secondary metabolites. Among the secondary metabolites, polyphenolic compounds are the most abundant in grape berries. Besides their important impacts on grape and wine quality, this class of compounds has beneficial effects on human health. Due to their antioxidant activity, polyphenols and phenolic acids can act as anti-inflammatory and anticancerogenic agents, and can modulate the immune system. In grape berries, polyphenols and phenolic acids can be located in the pericarp and seeds, but distribution differs considerably among these tissues. Although some classes of polyphenols and phenolic acids are under strict genetic control, the final content is highly influenced by environmental factors, such as climate, soil, vineyard, and management. This review aims to present the main classes of polyphenolic compounds and phenolic acids in different berry tissues and grape varieties and special emphasis on their beneficial effect on human health.

## 1. Introduction

In 2017 the worldwide grape production was 73 million tons and wine consumption was 244 hectolitres, which represents the grapevine as one of the main horticultural crops in the world [1]. The grapevine is part of the large genus *Vitis* which consists of 70 species divided into two subgenera, *Muscadinia* and *Euvitis*. Based on the geographical origin, *Euvitis* group can be divided into European (*V. vinifera* L.), East Asian (*V. amurensis* Rupr.), and North American species (*V. labrusca* L., *V. riparia* Michx., *V. berlandieri* Planch., etc.). One more group that can be considered as a specific group of grape cultivars includes interspecific hybrids, made by crossing *V. vinifera* cultivars with cultivars belonging to other *Vitis* species. In recent years there has been a growing interest in studying these cultivars [2,3,4,5] because of their quite different polyphenolic content than *V. vinifera* cultivars. Furthermore, they possess a level of resistance to some fungal diseases and pests.

*Vitis vinifera* has a long domestication history, and during the long cultivation period, thousands of grape varieties were developed, many of which are still in production [6]. Grapes are rich in primary and secondary metabolites which affect the quality. There are three distinct tissues in a grape berry (skin, pulp, seeds) which contain different groups of compounds, such as organic acids, sugars, volatile compounds, polyphenols, and phenolic acids. Polyphenolic compounds, together with phenolic acids, are a large group of plant secondary metabolites that can be divided based on structure. Polyphenols are defined as substances that possess multiple aromatic rings with one or more hydroxyl groups, while a phenol is any compound that contains an aromatic ring—regardless of the number of them—with one or more hydroxyl groups attached. Such a definition is not appropriate when referring to plant phenols because it would also include estrone and some carotenoids that are terpene by origin. In general, plant phenols and polyphenols refer to natural secondary metabolites derived from the shikimate/phenylpropane pathway and/or polyketide acetate/malonate pathway to form monomeric or polymeric forms, as chemically defined, and they are included in a very large number of physiological processes in plants [7,8]. According to this definition, stilbene and flavonoids are polyphenols, while phenolic acids are not polyphenols, so the terms phenols and phenolic compounds will be used hereinafter when phenolic acids, stilbene, and flavonoids need to be considered. One of the largest polyphenolic groups present in grapes is the flavonoids, which include anthocyanins, flavonols, and flavan-3-ols. Furthermore, grapes contain phenolic acids (hydroxycinnamic and hydroxybenzoic acids) and stilbenes. All these compounds play important roles in growth, reproduction, and defense reactions in plants. Based on the presence or absence of phenolic compounds, grape varieties can be divided into red and white varieties. Furthermore, these compounds have considerable influences on grape/wine quality and sensory characteristics—particularly astringency, bitterness, and color stability [9,10]. The final content and composition of phenolic compounds are influenced by multiple factors, such as grape variety, climate, soil, and growing conditions [11].

In the 1990s the phrase “French paradox” was established for observations that the French population suffered a lower incidence of coronary heart disease (CHD) despite the high intake of saturated fat. This paradox was attributed to high wine consumption [12]. Since then, many studies have focused on the benefits of moderate wine consumption and grape and wine compounds that have beneficial effects on human health [13,14,15,16,17]. It was shown that phenols have antioxidant activity, can be anti-inflammatory, anticancerogenic, and antibacterial, and can modulate the immune system. Hence, there is a growing interest in using these compounds in the food industry as natural additives, food coloring agents, or seasonings [18].

In the last ten years, numerous review papers and chapters have been published that provide overviews of the chemistry and biochemistry of polyphenols [11,19,20,21,22], their composition and content in grapes [23], their impacts on human health [24,25], and their interactions with different aspects of human health in more detail, such as anticancer [26], neuroprotective [26,27,28,29,30,31], anti-inflammatory [32,33,34], cardiovascular [35], and anti-diabetic [36] actions. This review aims to combine the most relevant data representing the composition and content of polyphenols in different varieties and organs of grapes, to present the most important techniques of qualitative and quantitative analysis of these compounds, and to give a comprehensive overview of the effects of polyphenols from vines on human health, including antioxidant activity, anti-inflammatory activity, cardiovascular protection, neuroprotective activity, anticancerogenic activity, and antimicrobial activity.

## 2. Extraction Techniques for Phenolic Compounds

The analysis of phenolic compounds consists of collecting the samples, sample preparation, instrumental analysis, and data processing. The most important step, which will affect the instrumental analysis and the data obtained, is sample preparation. This is the most critical and demanding step to improve the analysis concerning the matrix, the analyte, or both. Thus, the optimization of sample preparation reduces the total analysis time and avoids potential error sources, especially when working with samples at low concentrations [37].

A major step in sample preparation is the extraction process, including the choice of extraction technique, which is important for achieving good recoveries [8]. There are many extraction techniques for the recovery of phenols from grapes, such as solid–liquid extraction (SLE), ultrasound-assisted extraction (UAE), microwave-assisted extraction (MAE), enzyme-assisted extraction (EAE), matrix solid-phase dispersion (MSPD), supercritical fluid extraction (SFE), and pressurized liquid extraction (PLE). Details about above-mentioned techniques for extraction of phenolic compounds can be found elsewhere [21].

### 2.1. Solid–Liquid Extraction

Solid–liquid extraction is the most commonly used technique for the recovery of phenolic compounds. It works on a principle of the mass transfer phenomenon, where the analyte contained in a solid matrix is diffused into the extraction solvent. The extraction efficiency depends on process conditions and several factors should be considered: temperature, liquid-solid ratio, flow rate, and particle size [38]. The extraction process can be accomplished by various methods, such as maceration, shaking, or mixing; and using different solvents, such as methanol, ethanol, acetone, or their aqueous solutions. The pH value of the extraction solvent has a considerable influence on the extraction process, and extraction efficiency is higher in an acidic environment. To achieve acidic conditions, different acids can be added to the extraction solvent, such as formic acid, acetic acid, or hydrochloric acid [39,40]. Furthermore, the extraction time and temperature are factors that can affect the energy and time consumption, so it is desirable to optimize them. Increasing the extraction temperature leads to higher permeability of cell walls, higher solubility of phenols, and higher heat and mass transfer phenomena through the plant matrix [41]. Therefore, an increase of extracted solid can be observed at higher temperatures. Despite the positive effects of higher temperatures on the extraction yields, this cannot be increased indefinitely. It is considered that the stability of phenolic compounds and the denaturation of membranes can happen at temperatures higher than 50 °C [42]. Additionally, with the increase in temperature, the time of extraction can be considerably shortened. A higher solvent-to-solid ratio accelerates mass transfer phenomena due to a higher difference in concentration between the solid matrix and the bulk phase of the solvent. Hence, the extraction process progresses more rapidly, but the concentration of the phenolic compounds in extracts is lower, whereas the purity of the extracts may be poor due to the coextraction of non-desirable compounds [41]. Particle size is also an important factor that affects the extraction efficiency. The smaller particles increase the surface of the plant material in contact with the solvent and thus the rate of the mass transfer [8], which leads to a higher rate of extraction. Consequently, the extraction time can be shortened because the analyte must overcome a shorter path to reach the surface. Therefore, one of the pretreatment steps that should be considered is the comminuting or grinding of the raw material to obtain smaller particles [43].

SLE is an inexpensive, simple, and accurate extraction technique for the extraction of phenolic compounds from the grapevine. There is no need for expensive equipment. Depending on the method implementation, shaker or magnetic stirrer, a high throughput of the sample can be achieved. This technique is very suitable for routine analysis of phenolic compounds and commercial extraction when ethanol is used as the generally recognized as safe (GRAS) solvent. 

### 2.2. Ultrasound-Assisted Extraction

This novel method of extraction has been being used more and more lately as a stand-alone process or as a part of the stepwise procedure for valuable compounds extraction [44]. UAE works on a principle of partitioning the analyte between the solid matrix and the extraction solvent using high frequency (20–100 kHz) ultrasound. As the sound wave passes through an elastic medium it induces a longitudinal displacement of particles. If the ultrasound intensity is strong enough, it creates voids in the medium that make cavitation bubbles which are responsive to the ultrasonic effect. These cavitation bubbles are able to grow, and when they reach a critical size—an average of 150 µm—they collapse and release a large amount of energy. The high pressures (up to 2000 atmospheres) and temperatures (up to 5500 °C) involved in the process will destroy the cell walls of the plant matrix and their contents can be released into the medium [45]. UAE offers benefits such as greater penetration of the solvent into the cellular material, shorter processing and residence time, higher product yields and more accurate reproducibility, low solvent and emulsifier consumption, considerable savings in maintenance, and less energy needed for processing [44]. Moreover, UAE has proven to be a green technology. The application of ultrasound extends the range of solvent choice such that one may replace toxic organic solvents with generally recognized as safe (GRAS) solvents. Several environmental and economic benefits can be derived from reduced chemical usage, given that the UAE system is a simple, cost-effective, and efficient alternative to traditional extraction techniques [46,47,48].

Ultrasound showed to be a promising alternative for the extraction of phenolic compounds from grapes, allowing the production of extracts rich in these compounds. The most efficient extractions occurred at higher ultrasonic power, lower solvent-to-solid ratio, and higher acid concentration. In addition, ultrasound favored the extraction of rutin and quercetin, and promoted the hydrolysis of gallic, syringic, and *p*-coumaric acids [49]. Despite its efficiency and advantages, UAE is rarely applied for the recovery of phenolic compounds from grapes.

In comparison to SLE, the extraction time in UAE is shorter, but there are some issues during its implementation. This technique can be done in the ultrasonic bath and by specialized ultrasound probes. The latter one is a more expensive solution, and a small number of samples can be done in one run, with very accurate temperature control. During UAE in an ultrasonic bath, temperature control can be a problem because applied ultrasound waves heat the water in the bath. This method of implementation is suitable for short extraction and in most cases only one run at a time. Between the runs, there should be some time for cooling the water in the bath. Overall, this technique of extraction is suitable for the extraction of a small number of samples for research purposes. 

### 2.3. Microwave-Assisted Extraction

MAE is, like ultrasound, a green extraction method based on the direct impact on polar compounds [44]. The research on microwaves gave birth to the development of three types of techniques: microwave-assisted solvent extraction (MASE), solvent-free microwave extraction (SFME), and microwave extraction combining hydrodiffusion and gravity (MHG) [8].

This technique is based on microwave energy. The microwaves are part of the electromagnetic field in the frequency range from 300 MHz to 300 GHz. The extraction mechanism is supposed to involve three sequential steps: (1) the desorption of solutes from the active sites in the sample matrix under pressure and increased temperatures; (2) diffusion of extraction fluids into the sample matrix; and (3) release of the solutes from the sample matrix into the solvent [50]. The extraction process is influenced by many factors, such as extraction time, microwave radiation power, moisture content, particle size, type of solvent, and temperature. The most relevant factor is the type of extraction solvent. When selecting the solvent, it is important to consider three physical parameters: solubility of the analyte, dielectric constant, and the dissipation factor (degree of solvent heating by the action of microwaves). In general, the higher the dielectric constant and dielectric loss, the higher the capacity of the solvent to absorb microwave energy, which can lead to a faster rate of heating of the solvent with respect to the plant material. Additionally, by combining different solvents, the selectivity of the solvents for different compounds can be varied [51]. This was shown on the extraction of anthocyanins from grape skins [52], where the most considerable influence on the MAE was the extraction solvent, while other variables (time, temperature, microwave power, stirring) were less significant. Furthermore, when compared to SLE, there was a notable reduction in the extraction time from 5 h to 5 min. Additionally, MAE allowed treatment of up to 10 samples to be carried out simultaneously, which significantly reduced the analysis time per sample.

Microwave-assisted extraction with the use of modern technology can considerably improve extraction efficiencies. Moreover, it is a sustainable technology in achieving the objectives of green analytical chemistry with various advantages (high reproducibility, less solvent and energy consumption, more compact procedure, and greater purity of the final product) [51,53]. Like UAE, MAE is rarely used for the extraction of phenols from the grape. This technique is quite expensive because of the need for special equipment. The throughput of the samples is small because the number of places in the carousel is limited. Despite the short extraction time, the whole procedure is quite long because there is time needed for archiving experimental conditions (temperature), and post-extraction time for cooling and unpressurized the system. Overall, this technique of extraction is suitable for the extraction of a small number of samples for research purposes.

### 2.4. Enzyme-Assisted Extraction

Many phenolic compounds are located inside the cells, and to release the intracellular contents the cell wall must be degraded. Cell walls are highly complex and dynamic, composed of polysaccharides, phenolic compounds, and proteins, stabilized by ionic covalent linkages [54]. In grape skins phenolic compounds can be classified as cell-wall phenols, bound to polysaccharides by hydrophobic interactions and hydrogen bonds, and non-cell-wall phenols, including phenols confined in the vacuoles of plant cells and phenols associated with the cell nucleus [55]. Due to this complex matrix, it is necessary to use several enzymes, such as cellulose, pectinase, and tannase, to release the phenolic compounds [56]. The most commonly used enzymes are pectinases, a group of enzymes that catalyze the degradation of the pectin by depolymerization (hydrolases and lyases) and de-esterification (esterase) reactions. In commercially available enzyme preparations, the most common pectinases are pectin methylesterase (PME), pectin lyase (PL), and polygalacturonase (PG) [57]. Cellulases catalyze the breakdown of cellulose and are considered to be modular enzymes, composed of several subunits with specific functions and structures [58]. Tannase, or tannin acyl hydrolase, hydrolyzes ester and depside bonds of hydrolyzable tannins to produce gallic acid, glucose, and galloyl esters [59]. When using enzyme mixtures for the extraction, the fact that enzyme combinations can have positive or negative effects depending on the phenolic compound should be considered. It was shown that tannase enriches the phenolic extract in gallic and syringic acids, while cellulase enriches it in *p*-coumaric acid and malvidin-3-*O*-glucoside [56]. Pectinase affects the mechanical properties of berry skin, favoring the extraction of anthocyanins, oligomeric, and polymeric flavonols [60].

There are several advantages in using EAE: mild reaction conditions (low-temperature values, brief extraction period), the possibility of exploiting complete plant material, reduced number of stages, extraction yields of high quality, and bioavailability. As regulations regarding industrial extraction are getting stricter, EAE is continually gaining popularity in contrast to conventional extraction methods, which are considered environmentally hazardous [60]. EAE is an inexpensive, simple, and accurate extraction technique for the extraction of phenolic compounds from the grapevine. There is no need for expensive equipment. Depending on the method’s implementation, shaker or magnetic stirrer, high throughput of samples can be achieved. This technique is very suitable for routine analysis of phenolic compounds and commercial extraction because the enzymes applied are GRAS. 

## 3. Qualitative and Quantitative Analysis

Even though there is a very large number of studies on the topic, determining the composition and contents of different phenols is still a great challenge. Depending on the purpose of the research, different instrumental techniques for phenolic analysis are used. When it is necessary to estimate the total phenolic content, or the total contents of anthocyanins, flavan-3-ols, or flavonoids, different spectrophotometric methods can be applied. Various chromatographic techniques are used to determine the exact composition and content of phenolics in grape extracts, and nuclear magnetic resonance (NMR) and mass spectrometry (MS) are used to determine the structures of individual phenols [20].

Numerous spectrophotometric methods based on different principles have been developed to determine the content of phenolic compounds in different plant matrices and are used to determine the different functional groups present in phenol molecules. The simplest method for the rapid estimation of total phenolics content (TPC) in grape skin extracts is the measurement of absorbance at a wavelength of 280 nm (with a prior dilution of the sample). This method is based on the characteristic absorption of benzene phenol rings at 280 nm. The advantages of this method are the speed of analysis and good reproducibility. Some compounds, such as hydroxycinnamic acids, do not have absorption maxima at the specified wavelength. In addition to phenols, grape skin extracts may contain other benzene ring-containing compounds, which may lead to interference during analysis. Methods based on the determination of absorbances at wavelengths of 320, 360, and 520 nm, respectively, can be used to estimate the total contents of hydroxycinnamic acids, flavonols, and anthocyanins. The most commonly used method for estimating TPC is the Folin–Ciocalteu (FC) method based on the reducing properties of phenols. During the analysis procedure, phosphomolybdate is reduced in the presence of phenol under alkaline conditions to a blue-colored complex having a characteristic absorption maximum in the wavelength range from 725 to 765 nm. This method is not specific because the FC reagent also forms complexes with other compounds, such as proteins and other reducing agents (e.g., ascorbic acid), which may be contained in grape skin extracts. The results are expressed in equivalents of gallic acid (GAE) [20]. The total flavonoid content can be determined by a method based on flavonoid complexation reactions with aluminum ions, where complexes with an absorption maximum at 510 nm are formed in the presence of sodium nitrite and sodium hydroxide [61]. The content of total flavan-3-ol can be determined by the spectrophotometric method with vanillin as a reagent. The reaction between flavan-3-ol and vanillin takes place under acidic conditions to form adducts with an absorption maximum at 500 nm. The second method for determining the flavan-3-ol content is based on their reaction with 4-(dimethylamino)-cinnamaldehyde to form adducts with an absorption maximum at the wavelength of 640 nm [20,61]. There are several spectrophotometric methods for estimating the total anthocyanin content. One of the methods is based on the use of different pH values, using the property of anthocyanins that their wavelengths of absorption maxima change depending on the pH. By reducing the pH of the extract to values between 0.5 and 0.8, anthocyanins are converted to red-colored flavyl forms. Another way to determine the total anthocyanin content is to apply the method by bleaching with sodium bisulfite. The addition of this reagent to a sample of an extract containing anthocyanins leads to the formation of a colorless adduct of anthocyanin-sulfonic acid [20,39,40].

Electroanalytical techniques are groups of analytical techniques based on measurements of resistance, current, and potential, and all of them have in common that they take place in an electrochemical cell which is usually composed of two electrodes immersed in the corresponding electrolyte. These techniques can be divided into voltammetric, potentiometric, conductometric, coulorimetric, etc. Different voltammetric methods are the most commonly used among them, for phenol analysis. A common feature of all voltammetric techniques is that they involve applying a potential to the electrode and monitoring the current flowing through the electrochemical cell, so all voltammetric techniques can be described as functions of potential, current, and time. These techniques are considered active because the applied potential causes oxidation or reduction of electroactive species on the electrode’s surface, leading to changes in their concentration. Voltammetric techniques are a reliable tool for the rapid and inexpensive determination of phenolic compounds in various matrices. Methods have been developed that are precise and sensitive enough to be applied to samples containing very low analyte concentrations. Currently, these techniques are slowly replacing spectrophotometric methods for determining TPC. The great advantage of voltammetric techniques lies in the fact that it is not necessary to process the samples before the measurement, which reduces the duration of the analysis but also avoids errors associated with a large number of steps during sample preparation. Today, linear, cyclic, and differential pulse voltammetry techniques are most often used in practice. A glass or graphite electrode is used as the working electrode, while a silver electrode is used as the reference electrode [62,63,64].

High-performance liquid chromatography (HPLC) is most commonly used method to determine the precise composition and phenol content of grape extracts [65,66,67,68,69]. The efficiency of the HPLC method is influenced by the following factors: the composition of the stationary and mobile phases, the elution conditions, and the method of analyte detection. Reverse phase systems with octadecyl silicon dioxide (C18) are the most applied stationary phase to determine individual phenols in grape extracts [39,65,67]. In addition to this stationary phase, monolithic columns made of porous material in one piece can also be used. The use of such columns allows the faster flow of the mobile phase, which can be up to 5 mL/min, thereby shortening the analysis time and balancing the chromatographic system [69]. Today on the market there are special columns with a stationary phase made of nanoparticles and a solid core of silica gel coated with a porous layer of polymer, e.g., Core Shell columns. Using these columns can improve selectivity and resolution at the same time, thereby achieving a very low detection limit and high reproducibility [70]. Gradient elution using two solvent systems is the most commonly used elution method in phenol analysis. One of the solvents is polar, consisting of an aqueous solution of acetic, phosphoric, or formic acid, and the other solvent consists of acidified methanol or acetonitrile. The mobile phase flow rate ranges from 0.2 to 2.5 mL min^−1^ and the volume of the injected sample from 2 to 20 µL [52,65,66,67]. Phenolic compounds are good chromophores, and depending on the group to which they belong, have absorption maxima in a wide range of wavelengths from 280 to 530 nm, so a diode array detector (DAD) is most often used for their detection. Flavan-3-ols, stilbenes, and some hydroxycinnamic and hydroxybenzoic acids are also fluorophores that have characteristic excitation and emission spectra and can be determined using a fluorescence detector (FLD) which represents a very selective method of analysis. The HPLC–MS system is used to identify individual components in grape skin extracts, using mild ionization techniques such as electrospray ionization (ESI) and atmospheric pressure chemical ionization (APCI). A positive ESI is used to detect anthocyanins, whereas a negative ionization method is used to detect other groups of phenols. Each phenol has characteristic molecular ions and fragments based on which it is possible to determine its structure [71,72,73].

## 4. Phenolic Compounds in Grapevines

Grapes and leaves are a rich source of phenolic compounds, and in the grape berry, they can be found in the skin, pulp, and seed. These different tissues of the grape berry have different contents and compositions of phenolic compounds. The skin of a grape berry contains tannins and pigments, the pulp contains juice but no pigments, and seeds contain tannins. Biosynthesis of all phenolic compounds goes through the phenylpropanoid pathway from the amino acid phenylalanine, and two classes of compounds can be produced, flavonoids and stilbenes [11,74]. Phenolic compounds, as secondary metabolites, are frequently accumulated as glycosides. Thus, nonflavanoids accumulate in the vacuoles of mesocarp cells while flavonoids accumulate in the dermal cells of the skin tissue [75]. Many factors influence the biosynthesis of phenolic compounds, among which the most important is the genotype (cultivar). Other factors are related to environmental conditions in which the cultivar is grown, especially light, temperature, soil, and water availability. Additionally, the different management practices, such as irrigation, fertilization, yield management, and canopy management, can also have considerable influences on grape phenolic composition [76]. One more factor that has a considerable influence on determining the content of phenols in grapes is the analysis procedure, especially the extraction method used. Over the years many publications related to the analysis of grape and wine phenolic compounds have been published. Nonetheless, there is still no available standardized procedure for sample preparation, extraction, and analysis [15]. Furthermore, the content of phenolic compounds is expressed in different ways, which hinders the comparison of results. 

### 4.1. Phenolic Acids

Phenolic acids are divided into two main groups: benzoic acids, with seven carbon atoms, and cinnamic acids, containing nine carbon atoms. These compounds exist predominantly as hydroxybenzoic and hydroxycinnamic acids that may occur in either the free or the conjugated form [19].

**Figure 1:**
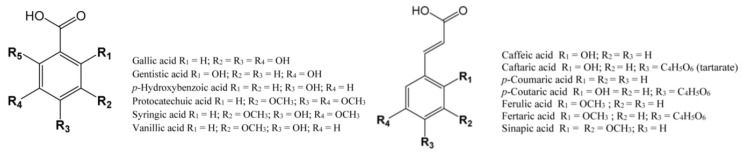
Structural formulas of grape phenolic acids [101].

Hydroxybenzoic acids are necessary for the synthesis of other compounds involved in the growth and development of a grape berry. In a grape berry, acids that can be found are gallic, vanillic, gentisic, protocatechuic, syringic, and *p*-hydroxybenzoic (Figure 1). Gallic acid is considered the most abundant benzoic acid and an important phenolic compound because it is a precursor of all hydrolyzable tannins and is part of condensed tannins [21]. Other hydroxybenzoic acids are components of lignins and higher content can be found in grape seeds. Table 1 presents the different grapevine cultivars and their contents of hydroxybenzoic acids in grapes and leaves. In most studies presented, gallic acid was analyzed, both in red and white grape cultivars. Even though the contents of vanillic and syringic acids are not analyzed in many studies, we can see that in most of the investigated cultivars their content is higher than the content of gallic acid.

Hydroxycinnamic acids are present in hypodermal cells, along with tannins and anthocyanins, and mesocarp and placental cells of the pulp. These compounds are the major class of phenolic acids in grape berries and white wine. In white wines, they can cause color browning under oxidation but also they are precursors of volatile phenolic compounds [19,89]. The most common acids are caffeic, *p*-coumaric, ferulic, and sinapic (Figure 1). These acids can be found in all grape tissues but predominantly in the vacuoles of pericarp cells. They differ by the type and number of substituents on the benzene ring. Furthermore, they can be found as cis or trans isomers, although grapes contain more trans isomers. These isomers can be converted enzymatically or through the action of light [19]. Hydroxycinnamic acids can occur in the free form or form of esters created with tartaric acid, sugars, or flavonoids. The list of hydroxybenzoic acid in Table 2 was chosen based on the literature data. Data about other acids, such as sinapic acid and 5-hydroxyferulic acid, are extremely rare. 

In the research of Liang et al. [82], more than 340 genotypes of *V. vinifera* were analyzed. Among other phenolic compounds, phenolic acids were analyzed. The authors observed that the contents of total hydroxybenzoic and hydroxycinnamic acids were higher in wine grapes than in table grapes. Additionally, the contents of these acids tended to increase as berry color became darker, although it was not statistically significant. In the group of hydroxybenzoic acids, the content of vanillic acid was generally higher than that of gallic acid and accounted for 70% of total hydroxybenzoic acids. In the group of hydroxycinnamic acids, caftaric acid was the most abundant and accounted for 74% of total hydroxycinnamic acids. The second most important was courtaric acid, which accounted for 22% of total hydroxycinnamic acids. The contents of hydroxycinnamic acids of some grapevine cultivars are presented in Table 2.

### 4.2. Stilbenes

**Figure 2:**
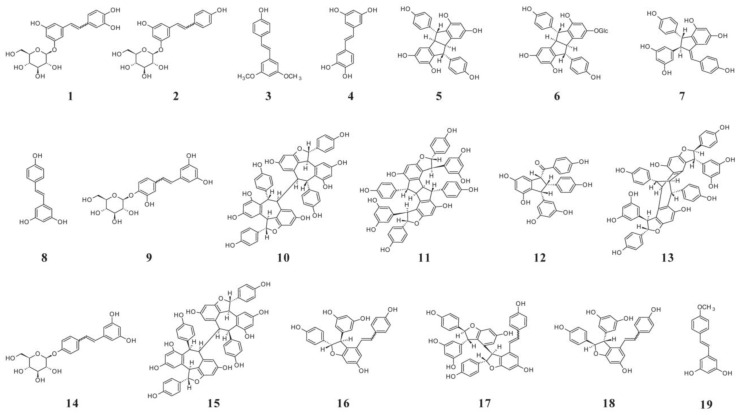
Structures of stilbenes in grape. (**1**) *Z*- and *E*-astringin; (**2**) *Z*- and *E*-piceid; (**3**) pterostilbene (3,5-dimethoxy-4′-hydroxystilbene); (**4**) piceatannol; (**5**) pallidol; (**6**) pallidol-3-*O*-glucoside; (**7**) parthenocissin A; (**8**) trans-resveratrol; (**9**) resveratroloside; (**10**) hopeaphenol; (**11**) ampelopsin H; (**12**) caraphenol B; (**13**) vaticanol C isomer; (**14**) resveratrol-4′-*O*-β-d-glucopyranoside; (**15**) isohopeaphenol; (**16**) *E*- and *Z*-ε-viniferin; (**17**) *E*- and *Z*-miyabenol C; (18) *E*- and *Z-δ*-viniferin; (**19**) *trans*-resveratrol-4′-methyl ether [11].

Stilbenes are a class of polyphenolic compounds that act as phytoalexins, protecting the berry from abiotic and biotic stress. In recent years there has been increased interest in this class of compounds, especially resveratrol, thanks to its beneficial effect on human health. They comprise two aromatic rings linked by an ethane bridge. The major content of stilbenes is located in grape skins but can also be found in grape seeds, stems, and leaves [102]. Stilbenes are formed through the phenylpropanoid and acetate-malonate pathway. The key enzyme is stilbene synthase (StSy) which catalyzes the formation of *trans*-resveratrol via condensation of 4-coumaroyl-CoA and malonyl-CoA [103]. The simplest stilbene is *trans*-resveratrol, while *cis*-resveratrol is a less stable isomer. Their 3-*O*-glucosides are known as *trans*- and *cis*-piceid, respectively. The more complex compounds formed by oligomerization and polymerization are known as viniferins. Viniferins are produced by oxidation of the basic stilbene by 4-hydroxystilben peroxidases. In grapes are found *α*-viniferin (a cyclic dehydrotrimer of resveratrol), *β*-viniferin (a cyclic dehydrotetramer of resveratrol), *γ*-viniferin (a more polymerized oligomer of resveratrol), *δ*-viniferin (an isomer of the resveratrol dehydrodimer), and *ε*-viniferin (a cyclic dehydrodimer of resveratrol) (Figure 2) [11]. Table 3 presents the contents of *trans*- and *cis*-resveratrol, and *trans*- and *cis*-piceid in the skins of different grapevine cultivars. In grape skins of Gamay and Chardonnay, in addition to *trans*-resveratrol, and *trans*- and *cis*-piceid, these cultivars contain 8.5 and 2.2 µg/g FW skin of ε-viniferin [104]. In grape cultivars Raboso Piave and Primitivo, considerable amounts of *E*- and *Z*-astringin and picetannol were found—in Raboso Piave, 106, 101.7, and 41.8 µg/kg grape; and in Primitivo, 884.2, 121.4, and 281.5 µg/kg grape [105]. 

The important compounds in the defense of grape berries against *Botrytis cinerea* are *ε*-viniferin, resveratrol, and piceid [106,107]. Furthermore, infection by *Plasmopara viticola* and UV radiation can induce the synthesis of *δ*-vinferin and *ε*-viniferin. Furthermore, the content of stilbene compounds depends on the ripening stage, and generally an increasing trend has been observed from *vérasion* to ripening stage [104]. Infection with a fungal disease, such as Botrytis, can induce the production of resveratrol. In research by Jeandet et al. [106] they observed that the distribution of stilbenes in an infected grape bunch strongly depends on the distribution of infected berries. The resveratrol content was predominantly present in grape berries close to the necrotic area and lesions caused by *Botrytis*. In non-infected berries, the content of stilbene compounds was very low. This localized response of phytoalexin production can help arrest the spread of *Botrytis* lesions.

### 4.3. Flavonoids

**Figure 3:**
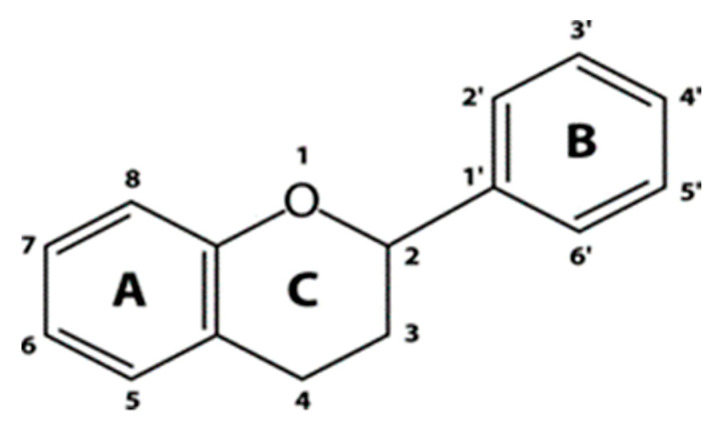
The general structure of flavonoids.

A great number of polyphenolic compounds in grapes are flavonoids. The general structure of flavonoids is two phenyl rings (A and B) and a heterocyclic ring (C) (Figure 3). Flavonoids are grouped into several classes that differ in the oxidation state of the C-ring, and they include anthocyanins, flavonols, and flavan-3-ols. They are synthesized from the combination of shikimic and polyketide pathways [110]. Flavonoids can occur either in free or in conjugated form, often esterified to one or two sugar molecules with at least one hydroxyl group [19,79]. Grape flavonoids are mainly localized in both the peripheral layers of a berry’s pericarp and in some layers of the seed coat. The inner thick-walled layers of hypodermis are where the major class of flavonoids is located, represented by anthocyanins, proanthocyanidins (tannins), and simple flavan-3-ols and flavonols [111].

#### 4.3.1. Anthocyanins

**Figure 4:**
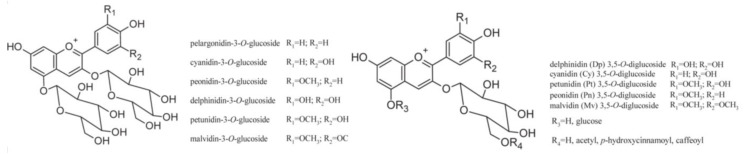
Structures of anthocyanins present in grapes [11].

Compounds that act as natural colorants present in the skin of red grape varieties are anthocyanins. These compounds are co-located with tannins in the thick-walled hypodermal cells of the skin [74] and are a glycosidic form of anthocyanidins. Grapes contain five out of 17 anthocyanidins, and these are delphinidin, peonidin, cyanidin, petunidin, and malvidin, which is usually the predominant anthocyanin in most red grapes [112]. In addition, pelargonidin was identified in berry skins of Cabernet Sauvignon and Pinot noir [113]. The anthocyanin structure has the typical C6–C3–C6 flavonoid skeleton, which contains one heterocyclic benzopyran ring (C ring), one fused aromatic ring (A ring), and one phenyl constituent (B ring) (Figure 4) [114]. In most *V. vinifera* grape berries, the glycosylation of anthocyanidins takes place exclusively at the 3-position by the activity of 3-*O*-glucosyltransferase, while in non-*V. vinifera* and their hybrids both 3- and 5-positions are glycosylated [115]. In 2015 evidence was provided confirming and explaining the existence of anthocyanidin-3-*O*-diglucoside in Cabernet Sauvignon grape berries. Until 2015 it was accepted that *V. vinifera* can contain only anthocyanidin-3-*O*-monoglucosides due to double mutation in the anthocyanin 5-*O*-glucosyltransferase gene which disrupts the enzymatic activity of the encoded protein [116]. However, the evidence showed otherwise, revealing a recombinant protein (designated as Vv5GT3) that can glycosylate the anthocyanidins on the 3-*O* and 5-*O*-positions [117].

The distribution of anthocyanins in different branches of a grape cluster is highly variable depending on many environmental and physiological factors. Anthocyanidins are synthesized in the cytoplasm but accumulate in vacuoles. During ripening, which affects anthocyanin biosynthesis, a two-stage accumulation is shown. In the first stage, there is a rapid increase of anthocyanins at the early stages of development, closely correlated with sugar accumulation, followed by the second stage characterized by slower accumulation [118]. Furthermore, the total amount of anthocyanins and the relative abundances of single anthocyanins are extremely variable among grapevine cultivars and are under strict genetic control [119]. The composition of anthocyanins for each variety is relatively stable, while absolute concentrations are strongly affected by both environmental and agronomical factors [120]. These can be seen in Table 4, where the distribution of anthocyanins is not identical in the case of same the same grape cultivar grown in different locations and growing seasons. The most abundant anthocyanin for almost all presented cultivars is malvidin-3-*O*-glucoside. 

Besides anthocyanidin-3-*O*-monoglucosides, there are also 3-*O*-acetylglucosides, 3-*O*-coumaroylglucosides, and 3-*O*-caffeoylglucosides present in different cultivars of grapevine. Their share is—aside from genotype—largely modified by the environmenatal conditions with dominant influences from light intensity and temperature. Anthocyanins are extracted from grape skin during vinification process in red wine production. The red wine hue and intensity differ among cultivars based on the total content of anthocyanins and shares of specific anthocyanin compouds. For same reason, by products of red grapes after red wine production are relatively poor sources of anthocyanins in comparison to fresh grape skins. There is an exeption in red grape byproducts from rosé wine production, in which the majority of anthocyanins are not extracted from grape skins. They can serve as a rich sourse of anthocyanins due to the fact that more than 15% of total red grapes produced are used for rosé wine production. 

After the transportation of anthocyanins to the vacuole, they adopt their distinct color, and this compartmentation decreases the feedback inhibition of cytosolic biosynthetic enzymes. What produces the red, violet, and blue coloration comes down to the presence and number of hydroxyl groups, methylations, and sugar moieties. Anthocyanins are red when conditions are acidic, colorless at pH 4.0, and purple above pH 4.5. Under alkaline conditions, blue color can be produced [75].

#### 4.3.2. Flavonols

**Figure 5:**
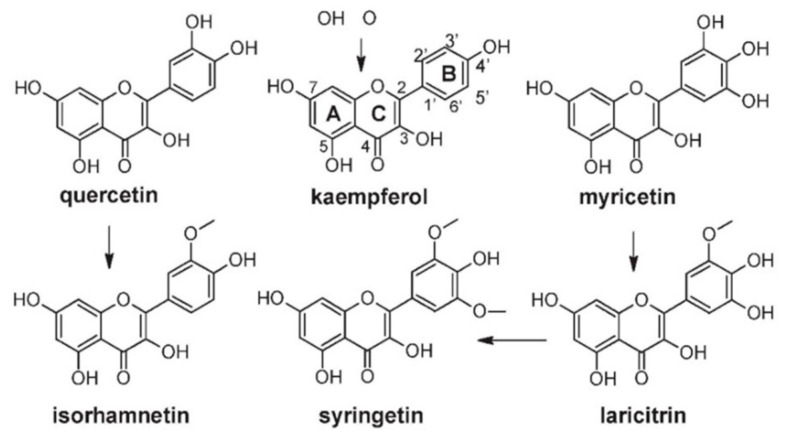
Structures of flavonol aglycones found in grapes [11].

Flavonols constitute a group of flavonoids that vary in color from white to yellow and are closely related in structure to flavones. The main representatives are kaempferol, quercetin, and myricetin. Common as well are simple derivatives such as isorhamnetin [128]. Moreover, the presence of both laricitrin and syringentin was also reported [65]. Flavonols have a double bond between positions 2 and 3 and a ketone group in position 4 of the C ring (Figure 5). Different sugars can be bound to position 3 of the flavonoid skeleton, producing glucosides, glucuronides, galactosides, and diglycosides [21]. Flavonol’s biosynthesis is closely related to that of anthocyanin and is under genetic control. Thus, like anthocyanins, they can be used in the taxonomical classification of both red and white varieties [120,129]. In the biosynthesis of flavonols, activity of flavonol synthase (FLS) activates enzymes that convert dihydriflavonols to flavonols. Between flowering and vérasion, the activity of FLS is commensurate with the pattern of flavonol accumulation in the berry. There are two distinct periods of flavonol synthesis in grape berries. The first period is around flowering and the second during the ripening of the developing berries [130]. Furthermore, it was shown that light and low temperatures have a synergistic effect on the expression of genes within the flavonoid biosynthesis pathway [131].

Flavonols are considered to act as UV- and photo-protectors because they absorb strongly at both UV-A and UV-B wavelengths. Thus, they can be mainly found in the outer epidermis of the skin [10]. Flavonols have also an important role in the stability of red wine color because they act as co-pigments with anthocyanins [132]. 

Table 5 represents the flavonol profiles of different grapevine cultivars. Castillo-Muñoz et al. [133] analyzed the flavonol profiles of 21 white grape cultivars. It was shown that the skins of white grape cultivars contain flavonols as 3-*O*-glycoside derivatives of only kaempferol, quercetin, and isorhamnetin. Quercetin-type flavonols dominated the flavonol profile of white grapes (60.8–90.7%), followed by kaempferol-type (8.8–38.3%) and isorhamnetin-type flavonols. Although isorhamnetin-type flavonols occurred as minor compounds, some cultivars (Gewürztraminer, Verdejo, Riesling) were characterized by relatively high and significantly different proportions of this compound. By analyzing 91 grape cultivars, among which 64 were red, Mattivi et al. [63] determined a pattern of the flavonols. The main compound in red grape cultivars was quercetin (12.34–87.76%), followed by myricetin (2.35–81.61%), kaempferol (0–17.52%), laricitrin (0–13.99%), isorhamnetin (0.42–11.88%), and syringetin (0–9.88%). 

#### 4.3.3. Flavan-3-ols

**Figure 6:**
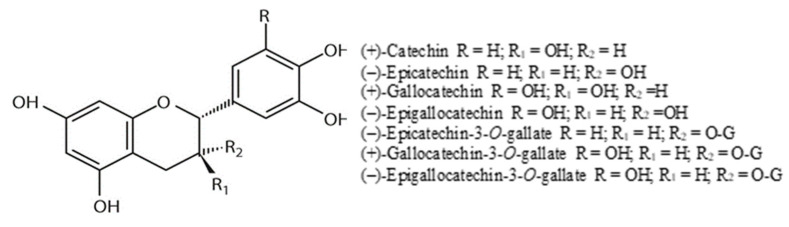
Structures of flavan-3-ol monomers present in grapes.

One of the principal classes of grape polyphenolic compounds is flavan-3-ols. In grapes, they are present as monomers or polymers called proanthocyanidins (PAs) or condensed tannins. The main monomers found in grapes are (+)-catechin, (−)-epicatechin, (+)-gallocatechin, (−)-epigallocatechin, and (−)-epicatechin-3-*O*-gallate (Figure 6) [73,135]. Proanthocyanidin structures vary depending upon the nature (stereochemistry and hydroxylation pattern) of flavan-3-ol units, the degree of polymerization, and the presence or absence of modifications [136]. PAs are mainly oligomeric and polymeric forms of (+)-catechin and (−)-epicatechin. The monomers are linked through C4–C6 or C4–C8 linkages (B type) with sometimes additional C2–O–C5 or C2–O–C7 bonds (A-type) [21,83]. PAs are the most abundant class of soluble polyphenolics in grape berries and can be found in hypodermal layers of the skin and the soft parenchyma of the seed coat between the cuticle and the hard seed coat. They are accumulated in specific vacuoles (tannin vacuoles) and act as deterrents to herbivores and fungi. Skin tannins have a much larger average size than seed tannins, and they contain epigallocatechin, whereas in seed tannins they are generally absent [74,75]. Flavan-3-ols in the grape berry start to accumulate before vérasion, especially during the early stage of development, and then decrease in concentration during ripening [137]. 

Table 6 depicts the contents of flavan-3-ols in the skins of different cultivars. In white grapevine cultivars, Semillon and Ugni blanc, the highest concentrations of procyanidins are present in the early stages of development. The major constituent of flavan-3-ols was (+)-catechin, along with procyanidin dimers B1, B4, and B6. During the maturation, the predominant dimers were B2 and B4 [138]. Cabernet Sauvignon is richer in low molecular weight procyanidins than Merlot. Additionally, dimers B2, B4, and B1 were found in both Merlot and Cabernet Sauvignon [139]. 

Flavan-3-ols are important in winemaking and affect wine’s sensory properties, contributing to astringency and bitterness. The intensity and duration of astringency can be attributed to structural characteristics (chain length, the stereochemistry of subunits, site of the bond between subunits). Bitterness is highest in the monomers and lowest in the trimers, whereas monomers are rated lower in astringency than the dimers or trimers [142,145]. Despite the differences in the content of flavan-3-ols depending on the cultivar, the contents of those compounds in the wine industry by-products are dominantly the results of the differences in grape processing technology of red and white wines. In the case of red wine production, which has longer contact of skins and seeds with juice (typically 7–10 days), their extraction is much higher than in the case of white wines. For the same reason, the resulting skins and seeds have higher contents of flavan-3-ols after white wine production, typically from white cultivars of grapevine. 

Astringency and bitterness are especially expressed in the case of young wines, especially in cases when grapes are not fully mature. This is common in cases of late-ripening cultivars and years with lower temperatures. Wines with higher levels of flavan-3-ols, despite their positive health influences, are usually not preferred by consumers, and wines with high contents are subjected to aging in wooden barrels, which results in their sedimentation.

## 5. Biosynthesis of Phenolic Compounds

**Figure 7:**
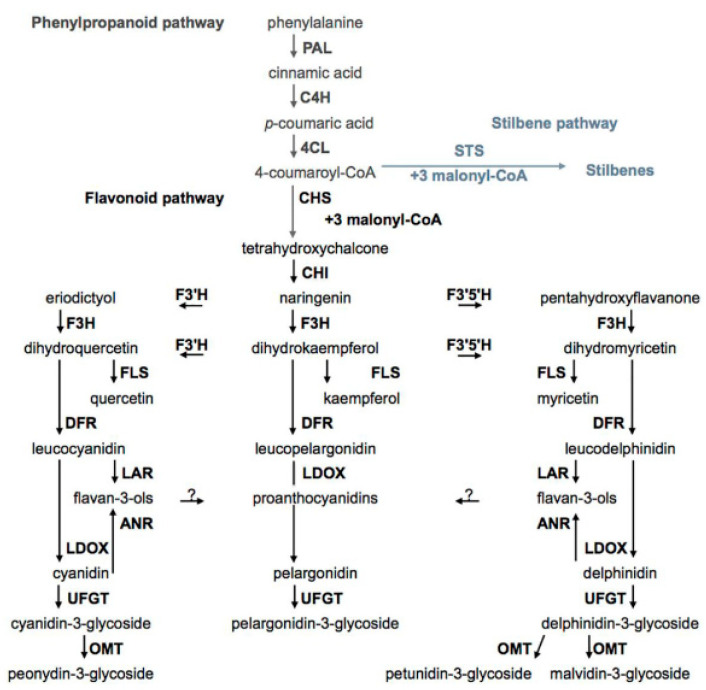
Biosyntheses of phenolic compounds. PAL—phenylalanine ammonia-lyase, C4H—cinnamate-4-hydroxyliase, 4CL—4-coumaroyl:CoA ligase, STS—stilbene synthase, CHS—chalcone synthase, CHI—chalcone isomerase, F3’H—flavonoid-3′-hydroxylase, F3′5′H—flavonoid-3′,5′-hydroxylase, F3H—flavanone-3-hydroxylase, FLS—flavonol synthase, DFR—dihydroflavonol reductase, LAR—leucoanthocyanidin reductase, LDOX—leucoanthocyanidin dioxygenase, ANR—anthocyanidin reductase, UFGT—UDP-glucose:flavonoid-3-*O*-glycosyl transferase, OMT—anthocyanin *O*-methyltransferase.

There are many review papers and book chapters which describe the biosynthesis of phenolic compounds in grapes [22]. In brief, the biosynthesis of phenolic compounds can be divided into several interconnected pathways. The first pathway, the phenylpropanoid pathway, includes the conversion of phenylalanine by three successive enzymatic reactions to 4-coumaroyl-CoA. The above-mentioned enzyme reaction is catalyzed by the following enzymes: phenylalanine ammonia-lyase (PAL), cinnamate-4-hydroxyliase (C4H), and 4-coumaroyl: CoA ligase (4CL). Phenolic acids are the products of modifications of intermediates of the phenylpropanoid pathway, and 4-coumaroyl-CoA, the end product of this branch, can be converted to an intermediate (tetrahydroxychalcone) for flavonoids’ biosynthesis by the action of chalcone synthase (CHS) or by stilbene synthase (STS) to an intermediate for stilbene biosynthesis of resveratrol (Figure 7) [111]. 

The second pathway is the flavonoid pathway. By this pathway biosyntheses of flavonols, flavan-3-ols, proanthocyanidins, and anthocyanidins occur. Chalcone isomerase (CHS) converts tetrahydroxychalcone to flavanone naringenin. Other flavanone eriodictyol and pentahydroxyflavanone are products of conversion of naringenin by enzymes flavonoid-3′-hydroxylase (F3′H) and flavonoid-3′,5′-hydroxylase (F3′5′H), respectively. By the activity of flavanone-3-hydroxylase (F3H), naringenin, eriodictyol, and pentahydroxyflavanone give dihydroxyflavonols, dihydroxykaempferol, dihydromyricetin, and dihydroquercetin, respectively. Enzyme flavonol synthase (FLS) catalyzes the conversion of three dihydroxyflavonols to the corresponding flavonols. The dihydroxyflavonols are also intermediates in the biosynthesis of flavan-3-ols and anthocyanins. In this branch of flavonoids biosynthesis, they are first converted to corresponding leucoanthocyanidines by the action of dihydroflavonol reductase (DFR). Leucoanthocyanidin reductase (LAR) converts leucoanthocyanidines to flavan-3-ols, and leucoanthocyanidin dioxygenase (LDOX) catalyzes the biosynthesis of the corresponding anthocyanidins. Anthocyanidins and flavonols are present in form of glycosides and this reaction is catalyzed by UDP-glucose: flavonoid-3-*O*-glycosyl transferase (UFGT) [11,22].

## 6. Grape Phenolics and Their Impact on Human Health

In the last few decades, there has been a growing interest in establishing a healthy diet and lifestyle that will maintain overall health and prevent stress-related diseases, such as cardiovascular disease (CVD), cancer, and diabetes [35]. Wine, grapes, and grape products have been consumed since ancient times. Grapes contain various nutrient elements, such as minerals, vitamins, edible fibers, and phytochemicals, among which the most important are polyphenolic compounds [24]. A lot of positive properties are attributed to phenolic compounds, such as antioxidant, anti-inflammatory, anticancerogenic, and antibacterial activity. 

### 6.1. Antioxidant Activity

Oxidative stress is an imbalance between antioxidant molecule production and the oxidative reactive species. This delicate balance aims to maintain a suitable redox potential. If the redox potential increases, there is a risk of disease occurrence, such as cardiovascular, metabolic, and neurodegenerative diseases. Thus, research in dietary intake that may reduce the incidence of these chronic diseases has become important [146].

Phenolic compounds can act as antioxidants mainly due to their redox potential, which allows them to act as reducing agents, hydrogen donors, and singlet oxygen quenchers [147]. Oxidation of lipoproteins, such as LDL, is an important step in the development of atherosclerosis. Therefore, dietary supplementation with antioxidant preparations containing polyphenols may reduce the risk of atherosclerosis [148]. In a study on healthy and hemodialysis subjects, the effect of concentrated red grape juice was studied. It was shown that grape juice consumption increased the antioxidant capacity of plasma, and reduced the concentration of oxidized LDL [149]. Leong et al. [150] studied the effect of Pinot noir juices on the protection of human intestinal Caco-2 cells from H_2_O_2_-induced stress. It was observed that the cell viability of Caco-2 under oxidative stress is positively correlated with the content malvidin-3-*O*-glucoside and total phenolics. Similar research was conducted by Wang et al. [151], wherein grape phenolic extract (GPE), rich in hydroxybenzoic acids, flavonols, and hydroxycinnamic acids, was used to observe the antioxidant protection of Caco-2 cells. The results showed that treatment with GPE was able to considerably reduce oxidative and protein damage, and reduce pro-oxidant-cytotoxicity. A study by Lingua et al. [152] on Caco-2 cells evaluated the effect of gastrointestinal (GI) digestion on antioxidant activity (AC) of white grapes, comparing them to those of their white wines. It was shown that the winemaking process modified the phenolic profile of grapes and on average only 12% of the TP (total polyphenols) content in grapes was present in wines. Furthermore, digestion reduced the phenolic content, with only 31% and 67% of native polyphenols from grapes and wines being potentially bioaccessible. It was also shown that cellular AC of non-digested and digested food was the same at the same polyphenol concentration. This indicates that changes in phenolic profile did not modify the bioactivity. 

A high-fat-diet (HFD) in mice can induce leaky gut syndrome combined with low-grade inflammation, which can increase reactive oxygen species (ROS) in the intestines and may contribute to dysbiosis and metabolic syndrome (MetS). A study on mice fed a HFD were administered GPE and β-carotene [153]. The results showed that GPE, rich in proanthocyanidins (PCA), decreased HFD-induced ROS. This reduction of ROS may benefit anaerobic and microaerophilic gut bacteria, such as *Akkermansia muciniphila*. This implies that the bloom of beneficial gut bacteria associated with improved metabolic status may be linked to the protective antioxidant activity of polyphenolic compounds. The influences of grape skin polyphenols may differ depending on their concentrations and intracellular ROS. In research on human intestinal cells (HT-29), in basal and stressed conditions, polyphenols showed pro-oxidant and anti-oxidant effects. In basal conditions, the pro-oxidant effect, corresponding to high ROS and low reduced glutathione (GSH) content, was due to the polyphenolic oxidation in cell culture with the production of hydrogen peroxide. On the other hand, in stressed conditions grape skin polyphenols showed anti-oxidant effects up to 1.3 × 10^−6^ µg/g and restored the stress-related GSH reduction [154].

Grape pomace, containing skins, seeds, and stems, is considered a waste in the winemaking process, and is usually discarded, despite its high content of bioactive compounds, including polyphenols. However, with the growing interest in sustainable winemaking, there is an awareness of using this waste and its bioactive compounds for different purposes, such as food coloring agents, nutritional additives, or as agents in pharmaceutical formulations. Cristea et al. [155] proposed using the grape marc extract for the development of a new food dye with antioxidant properties of natural origin. Grape pomace (GP) obtained from Nero d’Avola grape, rich in polyunsaturated fatty acids and polyphenols, presents strong antiradical and antiproliferative activity. The incubation of human hepatoma cell lines Hep-G2 with polyphenols showed a considerable effect on cell inhibition. The antioxidant activity was shown on fibroblasts HS-68, exposed to inducers oxidative stress. The incubation of cells for 24 h with polyphenols considerably inhibited cytotoxicity of the pro-oxidants [156]. Besides the grape pomace, the leaves can also be used as a source of many polyphenolic compounds. Maia et al. [157] showed that Pinot noir leaves are rich in phenolic compounds with antioxidant activity, such as caffeic acid, kaempferol, quercetin. All that should be very interesting for the pharmaceutical and food industries. 

### 6.2. Anti-Inflammatory Activity

Inflammation is a protective response of a tissue against cell injury, irritation, or pathogen invasion, and several environmental stress factors may cause inflammation [158]. The mechanism of proanthocyanidin’s (PAs) anti-inflammatory activity was investigated. Li et al. [159] evaluated grape seed proanthocyanidin’s anti-inflammatory activity in vitro and in vivo on rats and mice. It was observed that PAs had an inhibitory effect on paw edema in rats and ear swelling in mice. The author proposed that the major anti-inflammatory mechanism of PAs is related to its suppressive effect on the formation of inflammatory cytokines. Similar research was conducted on diet-induced obesity rats. The results showed that the daily consumption of PAs prevents inflammation in the adipose tissue, muscle, and liver, which might improve obesity-induced insulin resistance in these tissues [112]. Stilbenes are another class of polyphenols that exhibit anti-inflammatory activity, and plants containing stilbenoids have been used extensively in folk medicine [160]. Piceatannol is a stilbene compound that can be found in relatively low concentrations in wine but has strong antioxidant and anti-inflammatory activity. This was shown in a study where piceatannol inhibited mast cell-mediated allergic inflammatory reactions and their possible mechanisms, such as histamine release and MAPK pro-inflammatory cytokines. Resveratrol, the most known stilbene compound, also showed anti-inflammatory activity. It can have a protective effect in acute colitis, an inflammatory disorder, by reducing PGD2 production and the overexpression of COX-2. Additionally, it caused a considerable increase in TNBS-induced apoptosis [161]. Furthermore, resveratrol can suppress apoptosis and inflammatory signaling through its actions on the nuclear factor kappaB (NF-ĸB) pathway in human chondrocytes. These results suggest that resveratrol should be considered for the prophylactic treatment of osteoarthritis in human and companion animals [162]. 

### 6.3. Cardiovascular Protection

CVD is a major health problem worldwide affecting considerable proportions of the populations of developed countries. CVD is associated with high cholesterol levels in the blood—in particular, low-density lipoproteins (LDL). It is widely accepted that an excessive dietary intake of saturated fats and an unhealthy diet increases cholesterol and LDL levels in the blood [15,110,163] Thus, the research by Renaud et al. [12] that the French population suffered a relatively low incidence of coronary heart disease (CHD) despite the high intake of saturated fat, has sparked the interest of many researchers. The so-called “French paradox” was attributed to moderate wine consumption and its polyphenolic compounds. In a study on 69 male and female subjects, the effects of red wine and red grape extracts were studied. It was observed that moderate red wine consumption increased HDL-C by 11–16% and decreased fibrinogen by 8–15% compared with drinking water. Additionally, the red wine group had a mean weight loss of 0.11 kg. This suggests that moderate red wine consumption is associated with beneficial changes in blood lipids and fibrinogen that may reduce the CV risk factors [164]. Furthermore, alcohol, red wine, and polyphenolic grape extracts may inhibit platelet adhesion to fibrinogen which could attribute to the cardioprotective effects of prolonged and moderate alcohol or red wine consumption [165]. Consumption of capsules with polyphenolic extracts, containing 800 mg of polyphenolic compounds, lowers systolic blood pressure by 3 mmHg and diastolic blood pressure by 3 mmHg. Catechins and procyanidins are likely the class of flavonoids contributing to this blood pressure-lowering effect [166]. Chaves et al. [167] showed that acute consumption of grape extracts (GPE), equivalent to 1.25 cups of fresh grapes, considerably improved brachial artery flow-mediated dilation (FMD) within 3 h of consumption. Further GPE consumption twice a day for 3 weeks further improved FMD, and total antioxidant capacity in plasma was slightly increased. Additionally, the GPE consumption concomitant with high-fat meal prevented high fat-induced vascular endothelial dysfunction. A study conducted on rats during 14 months studied the long term impact of phenolic compounds (PC) on age-associated cardiac remodeling [168]. The three-month-old rats were daily treated till they were middle-aged with different doses of PC. It was observed that long-term daily consumption of PC preserves cardiac morphology and performance with less hypertrophy, inflammation, fibrosis, and cardiomyocyte apoptosis. These results showed that daily consumption of PC could have a positive effect on heart protection during aging. Poti et al. [169] performed a meta-analysis on data extracted from randomized controlled clinical trials involving subjects taking polyphenol-based supplements. The authors included 34 studies and performed a meta-analysis of the main parameters evaluated in the selected studies. The analyzed data showed high heterogeneity because of the differences in the treatment, in terms of formulation, dose, source, and identity of the evaluated polyphenol. Despite that, the overall analysis revealed a considerable effect of polyphenols in positively modulating the cardiovascular parameters considered. The polyphenols positively affected blood pressure, lowering systolic and diastolic pressure. The treatments lowered plasma levels of LDL-C while increasing HDL-C levels and FMD percentage. No considerable effects were detected for high-sensitivity C-reactive protein (hs-CRP). The authors concluded that even though these effects are statistically considerable, the detected differences are of modest size and their clinical benefits need further research.

### 6.4. Neuroprotective Activity

Phenols possess multiple neuroprotective effects that include protecting neurons from damage caused by neurotoxins, possessing the ability to prevent neuronal inflammation, and improving memory, learning, and cognitive functions. These effects can be achieved through two mechanisms. Phenols interact with important signaling pathways in the brain, causing inhibition of neurotoxin-induced apoptosis and promoting neuronal survival and differentiation. This group of compounds selectively acts on many protein kinases and the mode of signal transduction that regulate certain transcription factors responsible for the expression of certain genes. Polyphenols have a positive effect on the vascular system and thus affect the cerebrovascular circulation, which ultimately results in angiogenesis, neurogenesis, and changes in the morphology of neurons, thereby improving the ability to remember and learn. The formation of accumulations of amyloid fibrils is a common feature of many neurological diseases, such as Alzheimer’s (AD), Parkinson’s (PD), Huntington’s (HD), and prion diseases. The formation of amyloid fibrils is due to aggregation and deposition of misstructured proteins resulting from errors in axon signaling, inhibition of proteasomal activity, errors in DNA transcription, and increased levels of oxidative stress, which ultimately results in neuronal dysfunction. Phenols inhibit the formation of amyloid fibrils through special aromatic interactions, thereby preventing the formation of structured fibrils with a cytotoxic effect [170].

Youssef et al. [171] investigated the effect of grape seed and skin extract (GSSE) in a model of Parkison’s disease (PD). They discovered that GSSE has a neuroprotective effect on midbrain dopaminergic neurons by reducing apoptosis, the level of ROS, and inflammation. The GSSE protectd against neural loss and improved motor function in a model of PD.

The grape polyphenolic compounds can delay the initiation and/or hamper the progression of cognitive decline and AD. They can neutralize accumulated neurotoxins and inhibit apoptosis of neural cells caused by oxidative stressors and pro-inflammatory factors [172].

### 6.5. Anticancerogenic Activity

In the past few years interest in the concept and practice of chemoprevention as an approach to control cancer has increased greatly [173]. Polyphenolic compounds have also been shown to prevent the growth of cancer cells. In the study by Hsieh and Wu [174], the combination of epigallocatechin gallate, resveratrol, and *γ*-tocotrienol was used on breast cancer cells. This combination used at suboptimal doses elicited synergism in suppressing cell proliferation, modulating gene expression, and increasing antioxidant activity, as compared to each of the three phytochemicals added alone. In vitro and in vivo studies showed that resveratrol inhibits the growth of melanoma cells. When resveratrol was administered to mice, it reduced the growth of established melanoma cells and prolonged survival [175]. In a study by Nivelle et al. [176], resveratrol and various oligomeric derivatives were obtained from elicited grapevine cell suspensions. Four stilbenes (resveratrol, *ε*-viniferin, pallidol, and newly characterized dimer (6)) were recovered and assessed for their biological activity on the cell growth of two human skin malignant melanoma cancer cell lines (HT-144 and SKMEL-28) and a healthy human dermal fibroblast HDF line. The obtained results showed that resveratrol has the best anti-cancer properties because its efficiency against cancer cell viability is not affected by the presence of fetal bovine serum (FBS). Furthermore, resveratrol showed a tumor-specificity. Oligomers such as *ε*-viniferin and dimer (6) greatly reduced cancer cell viability, although this activity was considerably decreased in the presence of FBS. Anthocyanins can also have anticancerogenic activity. Cyanidin-3-*O*-glucoside, delphinidin-3-*O*-glucoside, and an anthocyanin-rich grape extract were used to treat colon and breast cancer cell lines. They were used alone or in the presence of a clinically used drug entacapone. Entacapone in combination with anthocyanins had a greater than additive effect on the growth inhibition of the colon cancer cells. Similar results were obtained with treated breast cancer cells, where entacapone enhanced the growth inhibitory activity of the anthocyanin extract. The drug also had antiproliferative effects when used as a single treatment. It was also shown that an important mechanism for growth inhibition is oxidative stress [177]. Cyanidin-3-*O*-β-glucopyranoside (C3G) was shown to have anti-proliferative and pro-differentiation properties. Treatment with C3G on two different prostate cancer cells displayed reduced cell viability and increased the levels of tumor suppressor [178]. Another study on prostate cancer cell lines was conducted using grape powder extract (GPE) [179]. The GPE treatment inhibited the cell viability and growth of prostate cancer cells only at 100 µg/mL concentrations. However, at low 1.5–15 µg/mL concentrations, GPE considerably reduced the colony formation and wound healing capabilities of prostate cancer cell lines. A natural red wine extract, containing polyphenols, flavonoids, and anthocyanins, was studied on the colon cancer cell line. The red wine extract can activate molecular pathways, involving Nrf2 signaling and the modulation of structural and signaling sphingolipid mediators that cooperate in promoting differentiation and reducing the proliferation of digestive tract cancer cells [180]. In another study, the peel and seed extract were incubated with human epidermoid carcinoma A431 cell lines to evaluate antiproliferative, apoptotic effects, and the morphological apoptotic changes induced by the extracts [181]. The study demonstrated that seed and peel extracts can inhibit the growth of A431 skin cancer cells by inducing cytotoxicity, generating reactive oxygen species followed by loss of mitochondrial membrane potential, and induction of apoptosis. Another study investigated the in vitro effects and putative action mechanisms of grape seed extract (GSE) on human breast cancer cells (MCF-7). The phenolic compounds were able to induce apoptotic cell death in MCF-7 cells at suitable concentrations. At the same time, GSE induced transient but considerable enhancement of GJIC in non-communicating MCF-7 cells and an early and dose-dependent re-localization of the connexin 43 (Cx43) proteins on the plasma membrane. The gap-junction-mediated cell-cell communications (GJIC) is a basal cellular function strictly related to the carcinogenic process and should be considered a target for potential chemotherapeutic compounds [182]. 

### 6.6. Antimicrobial Activity

One of the pharmacologically important properties of polyphenols and phenolic acids is their antimicrobial activity, i.e., antibacterial, antifungal, and antiviral activity. Their antibacterial activity has been the most researched. They can act through several mechanisms on numerous Gram-positive and Gram-negative bacteria. The mechanisms of antibacterial action of phenols include inhibition of intracellular enzymes, removal of substrate for bacterial growth, direct action on metabolic pathways such as oxidative phosphorylation or electron transfer, and prevention of metalloprotein synthesis through metal ion complexation. Inhibition of nucleic acid synthesis in certain bacteria is based on the inhibition of enzymes such as DNA gyrase and DNA topoisomerase. Due to their structure, phenols can interact with proteins, lipids, and certain enzymes of bacterial cell membranes, and thus cause changes in membrane functionality in the form of changes in fluidity and permeability, allowing the loss of protons, ions, and macromolecules, but also allowing other molecules to enter the cell, such as antibiotics. Oxidative phosphorylation is an important metabolic process for obtaining energy in the form of ATP molecules. Inhibition of only one of the enzymes of this process, such as NADH-cytochrome c reductase, inhibits the whole process, which inhibits the growth of bacteria. Some phenols can inhibit the human immunodeficiency virus (HIV) and in particular the pandemic strain of HIV-1 by preventing the virus from entering the host cell or preventing its replication by inhibiting key enzymes, such as HIV-1 reverse transcriptase and other DNA polymerases. Numerous phenolic compounds can also inhibit the activity of other viruses: herpes simplex virus, adenoviruses, respiratory syncytial virus, poliovirus, rabies virus, rotavirus, and Sindbis virus. Regarding those viruses, the polyphenols inhibit various DNA or RNA polymerases [183,184,185,186,187,188,189,190].

Bacteria are a large group of single-celled organisms that are microscopic in size and are some of the most widespread organisms in nature. They are found in water, air, and soil. They are an integral part of every food chain in nature. Bacteria can be found in all parts of the world, both in the tropics and in areas that are constantly under snow and ice. Many bacteria live in or on humans. They live in the intestinal flora, genitals, oral and nasal cavities, on the skin, and in other parts of the human body. Today, several thousand different types of bacteria have been described, some of which are very useful for humans, and about a hundred bacterial species can cause disease in humans. Pathogenic bacteria are those that can cause human disease, on their own or through their harmful products. Non-pathogenic bacteria can live in or on humans without causing any harm. Under certain circumstances, non-pathogenic bacteria can become pathogenic by penetrating tissues or organs that are not their natural habitats. Pathogenic bacteria are specific for a certain type of host and a special type of tissue. Some types of bacteria destroy the cells of their host. Many pathogenic bacteria produce toxins that are dangerous for the metabolism of the host cell. Pathogenic bacteria can be transmitted through water, food, air, coughing, sneezing, various secretions, and feces. Foodborne bacteria are causative agents of a great number of diseases and they have a great impact on human health and the economy. Among foodborne bacteria *Salmonella* spp., pathogenic *Escherichia coli*, *Shigella* spp., *Yersinia* spp., *Listeria monocytogensis*, *Staphylococcus aureus*, *Clostridium* spp., pathogenic *Bacillus* spp., *Vibrio* spp., and pathogenic *Campylobacter* spp., are of great importance [191,192]. 

The antimicrobial activity of different antibacterial agents can be expressed by the minimum inhibitory concentration (MIC) and the minimum bactericidal concentration (MBC). The MIC is defined as the lowest concentration of an antimicrobial ingredient or agent that is bacteriostatic (prevents the visible growth of bacteria). MICs are used to evaluate the antimicrobial efficacies of various compounds by measuring the effects of decreasing concentrations of the respective antibiotics/antiseptics over a defined period in terms of inhibition of microbial population growth. The MBC is the lowest concentration of an antibacterial agent required to kill a bacterium over a fixed, somewhat extended period, such as 18 h or 24 h, under a specific set of conditions. The MBC is identified by determining the lowest concentration of an antibacterial agent that reduces the viability of the initial bacterial inoculum by a pre-determined amount, such as ≥99.9%. The MBC is complementary to the MIC; whereas the MIC test demonstrates the lowest level of antimicrobial agent that greatly inhibits growth, the MBC demonstrates the lowest level of an antimicrobial agent resulting in microbial death. In other words, if a MIC shows inhibition, plating the bacteria onto agar might still result in organism proliferation because the antimicrobial did not cause death. Antibacterial agents are usually regarded as bactericidal if the MBC is no more than four times the MIC [193].

*Listeria monocytogenes* is a new pathogen in food microbiology. This bacterium is present everywhere in nature. Although it is isolated primarily from raw foods of plant and animal origins, it can also be present in cooked food as a result of subsequent contamination with dirty utensils and dishes (cooking removes it). Listeriosis (a disease caused by the bacterium *L. monocytogenes*) has very mild flu-like symptoms and very often goes unrecognized. However, infection with this bacterium is extremely dangerous for small children, pregnant women (in whom it causes abortion, fetal death, neonatal sepsis), the elderly, and immunocompromised persons, having relatively high mortality in those cases. The entry of bacteria into the body is oral, through food. Food from which *L. monocytogenes* is isolated is considered unhealthy. Anastasiadi et al. investigated the effects of grape seed extracts and grape steam extracts obtained from Voidomato seed and Mandilaria stems on the bacterium *L. monocytogenes*. The antimicrobial activity was expressed as MIC after 48 h of inoculation. The obtained values were 0.26 and 0.34 for grape seed extracts and grape steam extracts, respectively [194].

Bacteria species belong to the *Campylobacter* genus are the major cause of bacterial food-borne diarrheal diseases. This genus comprises 17 species, 14 of which are associated with human disease. Among them, *Campylobacter jejuni* and *Campylobacter coli* causes more than 95% of infections attributed to this genus. Silvan et al. investigated the effects of grape seed powder on the activity of nine *C. jejuni* strains (LP1, 118, CIII, CN1, CNL1, CNL2, NCTC 11351, NTCT 11168, and ATCC33291) and three *C. coli* strains (CNL4, LP2, and ATCC 43478). The extract, at the final concentration of 500 mg GAE/L, inhibits the growth of all examined bacteria strains. The inhibition of bacteria growth is in the range from 5.08 to 6.97 log CFU/mL. A resistant clinical isolate, *C. jejuni* LP1, was selected for the determination of MIC value (20 mg/L). The obtained MBC was 60 mg/L which is ten times lower than the most concentrated tested extract [195]. 

*Staphylococcus aureus* belongs to the family *Staphylococcaceae*. This family contains four genera, and members of these genera are Gram-positive, facultatively anaerobic, nonmotile cocci. Cocci can appear singly or in pairs, tetrads, short chains, or characteristic “grapelike” clusters. *S. aureus* produces enterotoxins responsible for food poisoning. The antimicrobial activity of muscadinia grape seed and grape skin extracts obtained from 10 cultivars against three *S. aureus* strains (ATCC 12600-U, ATCC 35548, and ATCC 29247) was determined. The average total phenol contents in grape seed extracts and grape skins extracts were 51.13 mg and 19.85 mg GAE/g, respectively. In the case of grape seed extracts average MIC values for ATCC 12600-U, ATCC 35548, and ATCC 29247 strains were 60.1, 54.8, and 59.6 μg/mL, respectively, while for grape skin extracts MIC values were 80.2, 70.7, and 76.7 μg/mL, respectively. These results indicate the correlation between total phenol content and antimicrobial activity. Higher polyphenolic content causes stronger inhibition of bacterial growth. The above-mentioned S. aureus strains are used for the antimicrobial study of the effectiveness of Carlos skins (CK) and seed (CS) extracts, and Noble skin (NK) and seed (NS) extracts. The phenolic contents of CK, CS, NK, and NS extracts were 39.0, 35.9, 58.8, and 58.2 mg GAE/g D.M., respectively. The MIC values for CS and NS (67 and 88 μg/mL) against ATCC 33548, ATCC 12600-U, and ATCC 29247 strains were, respectively, for NK, 113, 226, and 113 μg/mL, and for CK, 152, 304, and 152 μg/mL. The MIC values for pure compounds such as gallic acid, caffeic acid, catechin, ellagic acid, and quercetin were around 2500 μg/mL. The MIC values were determined for antibiotic compounds ampicillin, nalidixic acid, and streptomycin. Ampicillin had a MIC value lower than 1000 μg/L (5 μg/mL) only against ATCC 12600-U, while streptomycin had MICs of 20 μg/mL against ATCC 33548 and ATCC 12600-U strains. Nalidixic acid as a very strong antibiotic compound was very active against all analyzed strains with a value range from 20 to 156 μg/mL. Based on these results, grape seed extracts are very potent antimicrobial agents against three *S. aureus* strains—even stringer inhibitors than some antibiotics. The planktonic cell susceptibility to polyphenols and polyphenols inhibiting biofilm formation was determined by studying the ATCC 35548 strain and results are expressed as minimum biofilm inhibitory concentration (MBIC). The obtained MBIC value for inhibiting biofilm formation was within 2-fold lower than MICs, while MIBCs of preformed biofilm were 16-fold higher than MICs. The MICs for CS and NS against planktonic cells were 40 and 51 μg/mL, respectively; those against biofilm formation were 20 and 25 μg/mL, respectively; and those against preformed biofilm were 641 and 800 μg/mL, respectively. These findings indicate that GSE can inhibit bacterial biofilm formation at sub-MIC concentration [196,197]. 

Methicillin-resistant *Staphylococcus aureus* (MRSA) can cause nosocomial and community-acquired infections. The above-mentioned bacterium causes many diseases, such as mild skin infections, pneumonia, septicemia, and deep-seated abscesses. The effect of grape seed proanthocyanins extract (GPSE) on 43 MRSA strains was analyzed. All examined MRSA strains showed sensitivity against GPSE but with different levels of inhibition; thus, they can be divided into three groups based on the diameter of the inhibition zone: weakly sensitive (16 strains), moderately sensitive (15 strains), and highly sensitive (11 strains). At the concentration of 3 mg/mL of crude GPSE (the equivalent of 20.7 μg/mL flavonoid content), complete inhibition of all analyzed bacterial strains was observed. The GPSE has bactericidal activity because it disrupts the bacterial cell wall [198]. 

*Escherichia coli* is a Gram-negative, non-spore-forming, facultative anaerobe; a rod-shaped bacterium. It may or may not be mobile, but bacterial motility has principal roles in its adherence to surfaces and the potential formation of biofilms and gut colonization. *E. coli* encompasses a large and diverse group of bacteria. Most strains are harmless. Pathogenic strains can produce toxins. These strains are divided into six groups according to the mechanism of pathogenesis: enteropathogenic *E. coli* (EPEC), enterohemorrhagic *E. coli* (EHEC, also known as Shiga toxin-producing *E. coli*, STEC), enterotoxigenic *E. coli* (ETEC), enteroaggregative *E. coli* (EAggEC), enteroinvasive *E. coli* (EIEC), and attaching and effecting *E. coli* (A/EEC). The *E. coli* STEC group includes the following strains: O157:H7, 026:H11, O45:H2: O103:H2, O111:H2, O121:H19 and O145: NT. The major virulence factor of STEC strains is Shiga toxin (Stx). The effects of grape seed extract containing a minimum of 95% total flavonoids and 82% procyanidins on the seven above-mentioned STEC strains were studied. The MIC and MBC values were 2 mg/mL for the O26:H11 strain, and these values for all other strains were 4 mg/mL. The GSE content of 4 mg/mL effectively inhibited the growth of all tested strains, whereas 0.25–2 mg/mL GSE delayed bacterial growth when the level of inoculation was 5 × 10^5^ CFU/mL. A marked reduction in the swimming motility of all motile tested STEC strains can be achieved with GSE content of 0.125 mg/mL, while 4 mg/mL GSE inhibited the production of Stx in 103:H2, O111:H2, and O157:H7 strains [199,200]. 

Delgado Adamez et al. are collected grape seeds from Tempranillo grapes before and after vinification, and they got extracts from dry grape seed obtained before winemaikin (GSEJ) and dry grape seed obtained after winemaikin (GSEW), respectively. The antimicrobial activities of the GSEJ and GSEW were tested against three Gram-positive bacteria (910 *Listeria innocua*, 847 T *Brochothrix thermosphatica*, 910 *Staphylococcus aureus* subsp. *aureus*) and three Gram-negative bacteria (110 T *Pseudomonas aeruginosa*, 409 *Salmonella enterica* subsp. *enterica*, 45 *Escherichia coli*). In the seed extracts, dilutions of 100 and 50 µL/mL, inhibition for all analyzed bacteria was nearly 100% which means that both extracts were active against all examined bacteria. They were more effective against Gram-positive bacteria than Gram-negative bacteria. The latter bacteria have a lipopolysaccharide layer which is involved in the reduction of the sensitivity of those bacteria against extracts. The MIC was determined after 24 h of inoculation. This value for the GSEJ was 50 µL/mL for S. aureus subsp. aureus and *L. innocua* and 100 µL/mL for *B. thermosphatica* and *S. enterica* subsp. *Enterica*, while for *E. coli* and *P. aeruginosa* the MIC value exceeding 100 µL/mL In a case of GSEW, MICs for *S. aureus* subsp. *aureus* and *L. innocua* were 100 µL/mL, while for other bacteria MIC values were the same as for GSEJ extract [201].

The antimicrobial effect of grape pomace extract from Cabernet Sauvignon (132.2 mg of GAE/g) and Syrah (102.6 mg of GAE/g) is tested against two Gram-negative bacteria (*E. coli* ATCC 25922 and *Salmonella typhi* STH-2370) and two Gram-positive bacteria (*L. monocytogenes* ATCC 15311 and *S. aureus* ATCC 6538). Grape pomace extract had antibacterial activity against all the analyzed bacteria strains. At the concentration of 500 μg/mL extracts reach over 90% of inhibition for all studied bacteria apart from *S. Typhi* (70% of inhibition). In general, *S. aureus* and *E. coli* were the most sensitive bacteria exceeding 50% of inhibition at 62.5 μg/L concentration of extracts [202]. 

Jayaparkasha et al. are isolated grape seeds from Bangalore blue grapes and made an extract. The obtained extract is tested for antibacterial activity by pour plate method against *Bacillus cereus*, *Bacillus cougalans*, *Bacillus subtilis*, *S. aureus*, *E. coli*, and *P. aeruginosa*. The MIC values of GSE are determined by the number of colonies developed after incubation. The MIC was 900 ppm for *B. cereus*, *B. subtilis*, and *B. couagulans*, 1000 ppm for *S. aureus*, 1250 ppm for *E. coli*, and 1500 ppm for *P. aeruginosa*. Based on these values it can be concluded that GSE strongly inhibits grow of tested Gram-positive and Gram-negative bacteria [203]. 

*Helicobacter pylori* are the Gram-negative bacterium that is the etiological agent of peptic ulcers and gastritis. It is also associated with mucosa-associated lymphoid tissue lymphoma and gastric cancer. Martini et al. are examined the antimicrobial activity of Colorino, Sangiovese, and Cabernet Sauvignon grape extract, and isolated pure compounds resveratrol, epicatechin, myricetin, gallic acid, quercetin, quercetin-3-*O*-glucoside, and rutin on the CagA-G21 (not cytotoxic strain) and the CagA+ 10K (cytotoxic strain). Antimicrobial activity is expressed as the MBC. After 24 h of inoculation, the highest activity against the G21 strain had Colorino grape extract with an MBC value of 1.35 mg/mL, while Sangiovese and Cabernet Sauvignon had an MBC of about 4.0 mg/mL. Against 10K only Colorino grape extract was active with an MBC of 3.57 mg/mL. After 48 h of inoculation for all analyzed grape extract, MBC values against G21 strain were lower (0.89, 2.04, and 2.09 mg/mL for Colorino, Sangiovese, and Cabernet Sauvignon grape extract, respectively). In the case of the 10K strain, Colorino extract had the same value of MBC, while the values for Sangiovese and Cabernet Sauvignon were 4.08 and 4.18 mg/mL, respectively. The obtain MCB for individual compounds was lower in comparison with grape extracts. After 24 h of incubation, the MBC values for resveratrol, epicatechin, myricetin, gallic acid, quercetin, quercetin-3-*O*-glucoside were 68, 928, 122, 122,179 and 480 μg/mL, respectively for G21 strain, while for 10K strain were 84, 916, 930,490, 89 and 480 μg/mL, respectively. The other study conducted by Brown et al. is examined the effect of different grape extracts (red grape skin (RGS), white grape skin (WGS), black grape skin (BGS), muscadinia grape skin (MSN), muscadinia grape seed (MSD) and muscadinia grape synergy (MSY)) and resveratrol, ellagic acid and myricetin on H. pylori strains (G2-1, 26695, WV99, NB2-1, 1324P-1, D5251, D5131, D5178. D5136 and D5135). The antimicrobial activity of different grape extracts and individual compounds is expressed as MIC at 72 h post-inoculation. Among the studied strains, resveratrol, ellagic acid, and myricetin were active only against 26695 with the MIC values 12.5, 50, and 50 μg/mL, respectively. Grape extracts were active against all analyzed strains with MIC range from 256 up to 1024 μg/mL. Overall, MSN was most effective (MIC range, 256 to 512 μg/mL), followed by MSD (MIC range, 256 to 1024 μg/mL) and MSY (MIC range, 512 to 1024 μg/mL) [204,205].

*Porphyromodonas gingivalis* and *Fusobacterium nucleatum* are associated with periodontitis and other acute periodontal diseases. These bacteria are embedded in the plaque biofilms. The antibacterial activity of grape seed extracts (GSE) had MIC values 4000 and 2000 μg/mL for *P. gingivalis* and *F. nucleatum*, respectively determined 24 h after inoculation with bacteria. The determined MBC values for both bacteria were 8000 μg/mL. The GSE at the concentration of 2000 μg/mL had an inhibitory effect on the formation of the biofilm composed of *Lactobacillus*, *Streptococcus*, *Actinomyces*, *Fusobacterium*, and *Poryformonas* bacteria. The presented values indicate that GSE could be used in oral hygiene for the prevention of periodontitis [206]. 

Retroviral integrases (IN) belong to the family of polynucleotidyl transferases and are crucial for the pathogenesis of lentiviruses (HIV-1), thus they are good targets for antiviral agents. Pflieger et al. [207] are isolated 15 stilbenes from *V. vinifera* grapevine. Among them they identified 4 monomers (*E*-resveratrol, *E*-piceid, *E*-pterostilbene, and *E*-piceatannol), 6 dimers (leachianol F and G, *E-ε*-viniferin, *E*-*ε*-viniferinglucoside, *E*-scirpusin A, ampelopsin A, quadrangularin A, and pallidol), 1 trimer (*E*-miyabenol C) and 3 tetramers (hopeaphenol, isohopeaphenol, and vitisin D). The above mention compounds are potential inhibitors of HIV-1 IN, thus the activity was compared to those of well-characterized ratlegavir, an anti-integrase agent. The assay is performed on processed and unprocessed donor DNA. Leachianol G and F, quadrangularin A, *E*-scirpusin A, pallidol, *E*-piceid, and *E*-pterostilbene inhibited both activities, while *E*-*ε*-vinifern glucoside, *E*-resveratrol, and *E*-piceatannol inhibit integration from unprocessed DNA. Resveratrol dimer *trans-ε*-viniferin and tetramer r-2-viniferin can inhibit intestinal calcium-activated chloride channel and prevents diarrhea caused by rotavirus. The strongest inhibition of HIV-1 reaching 30–60% is observed with leachianol G and F in the concentration of 50 µM [208].

Hepatitis C virus (HCV) is a positive single-stranded RNA virus belonging to the *Flaviviridae* family. This virus contains 10 individual proteins, of which six are nonstructural (NS) proteins, NS2, NS3, NS4A, NS4B, NS5A, and NS5B. Nonstructural proteins are necessary for replication and other cellular processes, thus they are a potential target for antiviral agents. The HCV NS3 protein has protease and helicase activity so it is an indispensable component for the replication of HCV. Vitisin B has no effect on HCV entry in the host cell via the HCVpp system and does not affect the cleavage of polyprotein by the action of the NS3/NS4A protease, but it can negatively regulate HCV assembly/production, independent of its effect on the replication of HCV. The main inhibitory mode of action of vitisin B and *ε*-viniferin is the reduction of HCV protein production by inhibition of replication of the viral genome. These compounds specifically targeting the activity of NS3 helicase by direct binding and competing with its natural substrate, ATP. The anti-HCV activity of the vitisin B was EC_50_ = 6 nM and CC_50_ > 10 µM. [209,210].

*Herpesviridae* is a family of viruses consisting of 8 species each causes a distinct disease: humane cytomegalovirus (HCMV) herpes simplex virus 1 (HSV-1), herpes simplex virus 2 (HSV-2), varicella-zoster virus (VZV), human herpesvirus 6 (HHV-6), human herpesvirus 7 (HHV-7), human herpesvirus 8 (HHV-8), and Epstein–Barr virus (EBV). According to the Baltimore classification, they belong to the enveloped, nuclear replicating double-stranded DNA viruses. These viruses rely on the host machinery for DNA replication and transcription via RNA [211]. The HCMV is a lymphotropic virus that replicates and establishes latency in tissues associated with the lymphatic system. This virus is a causative agent of mononucleosis. Selective inhibition of HCMV replication can be achieved by inhibition of cellular p38 mitogen-activated protein kinase. Entry and/or signal transduction via epidermal growth factor receptor (EGFR) and downstream activation of phosphatidylinositol 3-kinase (PI3-K) and effectors such as p70S6K and Act are the initial events in the replication of HCMV. The major IE gene promoter (MIEP) of HCMV which is necessary for viral genome expression is activated by transcription factors NF-κB and Sp1. Resveratrol inhibited autophosphorylation of the EGFR and PI3-K signaling and the overall result is the inhibition of viral DNA replication. It also fully inhibits the initial phase of NF-κB and Sp1 activation and thereby reducing activities of DNA binding to those proteins. Resveratrol blocks the appearance of immediate-early, early, and late viral proteins of HCMV. Overall, this stilbene act early in the replication cycle of HCMV, no later than 4 h postinfection. Inhibition of the HCMV was IC_50_ = 1–2 µM [212]. The HSV-1, HSV-2, and VZV infect nervous tissue (neurotropic viruses). Herpes simplex virus 1 and 2 are causative agents of recurrent facial and genital herpetic lesions. The genome of the HSV virus is very complex and encodes nearly 100 transcripts. The productive replication cycle is composed of different stages: immediate-early (no viral protein synthesis), early (no DNA replication), and late (after viral genome replication). The entry of the virus to the cell requires interaction between viral membrane glycoproteins and cellular receptors, herpesvirus entry mediators (HVEM) which are related to the proteins that interact with the tumor necrosis factor (TNF). Host nuclear transcription factor NF-κB can be activated by different stimuli such as inflammatory cytokines, growth factors, and bacterial or viral infections. This factor is activated in response to HSV infection and at least two immediate-early herpes proteins, ICP4 and ICP27. The above mention factor interacts directly with the genome of the HSV virus via binding to the promoter of an immediate-early gene, ICP0. Resveratrol suppresses activation of NF-κB in cells infected by HSV-1 and HSV-2. This compound also negatively affects the synthesis of mRNA for ICP0 and ICP4 genes (immediate-early genes), and HSV DNA polymerase and single-stranded DNA binding protein (ICP8) (early genes). This led to the considerable inhibition of od DNA synthesis and late gene activation. Resveratrol in the concentration of 219 µM reduces viral titer by 99.9%. Overall, resveratrol inhibits early phases of HSV replication and has a great impact on the later viral cycle phases [213]. Varicella-zoster virus causes two distinct clinical entities: varicella (chickenpox) and herpes zoster (shingles). The genome of VZV contains double-stranded DNA with 125kb. Resveratrol does not affect the attachment of the virus to the host cell. It interferes with the first stage of the replication of VZV by suppression of activation of an immediate-early gene, ie62. IE62 protein is an essential regulatory protein of VZV. This protein structurally and functionally is similar to the ICP4 from HSV and regulates the expression of the viral genome. Resveratrol by reducing the level of mRNA affects the synthesis of IE62. In the concentration of 219 µM inhibit VZV replication with the lower limit of plaque assay of 10 pfu/mL [214].

Influenza A virus belongs to the family of orthomyxoviruses. It contains a segmented genome composed of minus-strand RNA. Like other orthomyxoviruses, the influenza A virus has enveloped virions with spikes. There are two different types of spikes composed of hemagglutinin (HA) protein and neuraminidase (NA) protein. Characteristics of the above-mentioned virus are that it has a viral ribonucleoprotein (vRNP) made of eight segments of (−)RNA associated with nucleocapsid proteins and enzymes, PB1, PA, and PB2 which are components of RNA-dependent RNA RNA polymerase (RdRp). The viral RNA encodes 17 influenzas A proteins, six of which are phosphorylated. During influenza virus infection, respective kinases cascades are activated including p38MAPK and JNK pathways and Raf/MEK/ERK cascade. Palamara et al. [215] in-depth investigated the effect of resveratrol on the replication of the virus in vivo and in vitro. Based on obtained results they concluded that resveratrol blocks nuclear-cytoplasmic translocation of vRNP complexes decreases expression of late viral proteins, and an inhibition of cellular protein kinase C (PKC) activity and its dependent pathways. Resveratrol inhibits the expression of M1 and HA on the post-transcriptional level by blockage of the nucleocytoplasmic translocation of vRNPs. Resveratrol inhibits the activities of PKC, and thus effects MPAK pathways (p38MPAK and JNK). Resveratrol in a concentration of 40 µg/mL completely blocks viral replication. Administration of resveratrol to mouses infected with influenza A virus considerably improves their survival and decreases pulmonary virus titers.

The severe acute respiratory syndrome coronavirus 2 (SARS-CoV-2) the causative agent of Coronavirus Disease 2019 (COVID-19), first emerged in 2019 and triggered a global pandemic affecting more than 215 countries. As of October 2020, there were more than 42,000,000 confirmed cases and more than 1,100,000 deaths. At this moment, there are no specific antiviral drugs or vaccines available for the treatment of COVID-19. This virus belongs to the *Coronaviridae* family of viruses which are enveloped, single-stranded positive-sense RNA viruses. The genome is ≈29.9 kb long, composed of 12 functional open reading frames and coated with nucleocapsid (N) protein, and enclosed by a lipid bilayer consisting of the three membrane proteins: spike (S), membrane (M), and envelope (E). The genome encodes 12 proteins, such as main protease (M^Pro^), RdRp, RNA binding N terminal domain (NTD) of nucleocapsid protein (N protein), viral ion channel (E protein), 2′-*O*-RIBOSEMethyltransferase, and human angiotensin-converting-enzyme 2 receptor (hACE-2) for entry into target cells. The above-mentioned proteins are the key targets for drug development. Some studies confirmed that kaempferol, quercetin, rutin, catechin, epigallocatechin, epigallocatechin gallate, ferulic acid, caffeic acid, *δ*-viniferin, and delphinidin-3-*O*-glucoside inhibit ACE-2 receptor and can potentially inhibit the entry of the virus into the target cells. Malvidin-3,5-*O*-diglucoside, 

*δ*-viniferin, delphinidin-3-*O*-glucoside, kaempferol, malvidin-3-*O*-glucoside, myricetin, and procyanidins have a high affinity for binding to M^Pro^, RdRp, and hACE-2 so could inhibit viral replication in host cells [216,217,218,219].

## 7. Conclusions and Future Perspectives

Grape phenolic compounds are a large and diverse class of compounds found in grapes. Many factors influence the final contents and composition of phenols in grape, among which are genetic factors, environmental ones, and cultural practices. Phenolic compounds have important roles in plants, as they act as phytoalexins, UV-protectants, and decrease the reactions caused by abiotic and biotic stresses. Furthermore, they are important in winemaking, as they influence wine color and pigmentation, moth-feel, bitterness, and astringency. Additionally, the consumption of wine and fresh grapes and its products have shown the positive influence of polyphenolic compounds on human health. Different studies showed antioxidant, anti-inflammatory, neuroprotective, anticancerogenic, and antimicrobial activity of phenols. Thus, it would be beneficial to include fresh grapes and their products, along with moderate wine consumption, in our diet.

In this review, a comprehensive presentation of phenolic compounds in grapes and grape leaves is given. It includes extraction techniques, methods of analysis, composition, and contents of phenols in individual varieties with a special emphasis on their impacts on human health. 

Despite the differences in expression in different studies related to the content of phenolic compounds in grapes and grapevine leaves, it is clear that there is huge variability in the contents of numerous phenolic compounds related to specific cultivars, but for the same reason, it is difficult to compare this results. Some efforts should be made in the future to obtain more comparable datasets. 

There are numerous extraction techniques for the isolation of phenolic compounds from the grapevine. The choice of the technique is strongly dependent upon available laboratory equipment and the goal of the extraction. The highest throughput of samples can be achieved by the application of SLE and EAE. These techniques are also suitable for the commercial extraction of phenolic compounds because GRAS solvents can be used. 

Non-communicable diseases such as cardiovascular disease, diabetes, cancers, and many neurodegenerative diseases are the leading causes of death in developed countries. A large amount of money is spent on the treatment of these diseases, which makes them a major economic problem. Synthetic drugs available on the market very often have several side effects, and their long-term use very often harms organs that were not originally affected by the disease. Polyphenols isolated from the vine are not cytotoxic, and numerous studies have shown that they have a positive effect on the cardiovascular system, and neuroprotective and anticancerogenic activities. An increase in the number of studies for the treatment of these diseases is necessary to determine their effects and to become approved drugs by the competent authorities such are FDA and EMA.

Grapevine extracts and individual polyphenolic compounds isolated from grapevines show strong antimicrobial activity in terms of antibacterial and antiviral action. These properties of the above-mentioned compounds could have a strong impact on humanity. In the last ten years, an increasing number of different strains of bacteria that are resistant to existing antibiotics have been discovered. They have greatly increased mortality from certain bacterial infections. The development of new antibiotics is a very time consuming and expensive process with an uncertain outcome. Therefore, research on natural compounds with antimicrobial activity constitutes a turning point in solving problems in the treatment of diseases caused by resistant strains. Numerous studies have shown that grape polyphenols can effectively inhibit the growth of certain strains of bacteria, but also act synergistically with antibiotics on otherwise resistant bacteria. Compared to the number of effective antibiotics available on the market, the number of antiviral drugs is small. The action of certain antiviral drugs is generally limited to a small number of virus strains. Numerous studies confirm that polyphenols isolated from vines have an antiviral effect on several viruses. Due to climate change and many other factors of today’s lifestyle, the emergence of new pandemic viruses is to be expected in the future. Therefore, new research on the antiviral activity of polyphenols is necessary.

Grapevine genetic resources represent a huge and mostly unexplored source of different phenolic compounds, with the majority of results being available only on a small and limited number of cultivars that are in the focus of the grape and wine industry. Given that there are more than 5000 different cultivars of grapevine still existing in grapevine repositories around the world, huge efforts are still needed in the future to obtain full insights into grapevines as a source of phenolic compounds. Taking into account that in the majority of studies a synergistic effect of individual compounds present in the grape extracts is observed, and that these contents are not very high, it can be concluded that the grape is a very potent source of polyphenolic compounds with effects on human health. 

## Figures and Tables

**Table 1:** Content of hydroxybenzoic acid in the grapes and leaves of different grapevine cultivars.

Cultivar (Country of Origin *)	Gallic Acid	Protocatechuic Acid	*p*-Hydroxybenzoic Acid	Gentisic Acid	Vanillic Acid	Syringic Acid	Analysis Method **	Ref.
	**Grapes–Red Cultivars**							
Alphonse Lavallee (FRA)	0 ^m^	0 ^m^	n.a. ***	n.a.	n.a.	n.a.	1	[77]
Azal Tinto (POR)	3.4 ^m^	n.a.	n.a.	n.a.	n.a.	2.5 ^m^	2	[78]
Borracal (ESP)	4.6 ^m^	n.a.	n.a.	n.a.	n.a.	13 ^m^	3	[78]
Cabernet Sauvignon (FRA)	11.93 ^a^	n.a.	n.a.	n.a.	n.a.	n.a.	4	[79]
	21.5–27.7 ^d^	n.a.	n.a.	n.a.	n.a.	n.a.	4	[80]
	3.66 ^i^	0.44 ^i^	1.60 ^i^	6.74 ^i^	n.a.	n.a.	5	[81]
	n.a.	n.a.	n.a.	n.a.	0.003 ^l^	n.a.	6	[82]
	82.6 ^o^	15.4 ^o^	n.a.	n.a.	108.5 ^o^	120.9 ^o^	3	[83]
Cabernet Franc (FRA)	16.7–16.9 ^f^	n.a.	n.a.	n.a.	2.2–2.5 ^f^	n.a.	4	[83]
	8.76 ^i^	0.43 ^i^	0.44 ^i^	0 ^i^	n.a.	n.a.	5	[84]
Dornfelder (GER)	n.a.	n.a.	n.a.	n.a.	0.011 ^l^	n.a.	6	[82]
Espadeiro (POR)	5.1 ^m^	n.a.	n.a.	n.a.	n.a.	0 ^m^	2	[78]
Merlot (FRA)	19.3–40.0 ^d^	n.a.	n.a.	n.a.	n.a.	n.a.	4	[81]
	3.66 ^i^	0.48 ^i^	0 ^i^	0 ^i^	n.a.	n.a.	5	[84]
	3 ^j^	n.a.	n.a.	n.a.	n.a.	n.a.	4	[85]
	125.6 ^o^	51.7 ^o^	n.a.	n.a.	197.9 ^o^	121.8 ^o^	3	[78]
	66.6 ^m^	328.7 ^m^	n.a.	n.a.	n.a.	n.a.	1	[77]
Negroamaro (ITA)	7.3 ^m^	42.0 ^m^	n.a.	n.a.	n.a.	n.a.	1	[77]
Pedral (POR)	2.2 ^m^	n.a.	n.a.	n.a.	n.a.	0 ^m^	2	[78]
Pinot Noir (FRA)	38.4 ^g^	n.a.	n.a.	n.a.	n.a.	32.8 ^g^	4	[84]
	2.42 ^i^	0.40 ^i^	1.42 ^i^	5.64 ^i^	n.a.	n.a.	5	[84]
	n.a.	n.a.	n.a.	n.a.	0.014 ^l^	n.a.	6	[82]
Pinot Gris (FRA)	16.1 ^g^	n.a.	n.a.	n.a.	n.a.	20.2 ^g^	4	[84]
	2.34 ^i^	0.54 ^i^	0 ^i^	5.90 ^i^	n.a.	n.a.	5	[84]
Plavac Mali (CRO)	n.a.	n.a.	n.a.	n.a.	0.047 ^l^	n.a.	6	[82]
Primitivo (CRO)	0 ^m^	13.4 ^m^	n.a.	n.a.	n.a.	n.a.	1	[77]
Prokupac (MNE)	3.90 ^i^	0.52 ^i^	0 ^i^	7.15 ^i^	n.a.	n.a.	5	[84]
Sangiovese (ITA)	4.80 ^i^	0.44 ^i^	0.30 ^i^	10.16 ^i^	n.a.	n.a.	5	[84]
Syrah (FRA)	0.23–0.33 ^c^	n.a.	n.a.	n.a.	n.a.	n.a.	4	[80]
	5.85 ^i^	0.44 ^i^	0.77 ^i^	8.74 ^i^	n.a.	n.a.	5	[84]
	16.45 ^k^	n.a.	n.a.	n.a.	n.a.	n.a.	4	[86]
Touriga (POR)	n.a.	n.a.	n.a.	n.a.	0.015 ^l^	n.a.	6	[82]
Tempranillo (ESP)	0.40 ^b^	n.a.	n.a.	n.a.	n.a.	n.a.	2	[84]
Vinhao (POR)	2.7 ^m^	n.a.	n.a.	n.a.	n.a.	8.9 ^m^	2	[78]
Vranac (SRB)	0.41 ^h^	n.a.	n.a.	n.a.	n.a.	n.a.	4	[84]
	**Grapes–White Cultivars**							
Albarino (ESP)	1.19 ^n^	n.a.	2.32 ^n^	n.a.	n.a.	n.a.	7	[87]
Chardonnay (FRA)	2.78 ^i^	0.49 ^i^	0 ^i^	5.87 ^i^	n.a.	n.a.	5	[84]
	5 ^j^	n.a.	n.a.	n.a.	n.a.	n.a.	4	[85]
	n.a.	n.a.	n.a.	n.a.	0.017 ^l^	n.a.	6	[82]
	29.4 ^o^	1.51 ^o^	n.a.	n.a.	249.8 ^o^	42.7 ^o^	3	[78]
Malvasia Fina (POR)	5.7 ^g^	n.a.	n.a.	n.a.	n.a.	5.5 ^g^	4	[84]
Pinot Blanc (FRA)	18.3 ^g^	n.a.	n.a.	n.a.	n.a.	17.2 ^g^	4	[84]
Riesling (GER)	2.44 ^i^	0.35 ^i^	0 ^i^	0 ^i^	n.a.	n.a.	5	[84]
Sauvignon blanc (FRA)	4.25 ^i^	0.55 ^i^	0 ^i^	1.47 ^i^	n.a.	n.a.	5	[84]
	n.a.	n.a.	n.a.	n.a.	0.030 ^l^	n.a.	6	[82]
	16.8 ^o^	2.10 ^o^	n.a.	n.a.	264.7 ^o^	95.2 ^o^	3	[78]
Verdelho (POR)	4.6 ^m^	n.a.	n.a.	n.a.	n.a.	0 ^m^	3	[78]
Vermentino (ITA)	11.9 ^o^	0 ^o^	n.a.	n.a.	74.9 ^o^	55.3 ^o^	3	[78]
Viognier (FRA)	10.2 ^o^	0 ^o^	n.a.	n.a.	153.9 ^o^	33.6 ^o^	3	[78]
Welschriesling (NN)	4.57 ^i^	0.44 ^i^	0 ^i^	0 ^i^	n.a.	n.a.	5	[84]
	**Leaves**							
Cabernet franc (FRA)	4.72 ^r^	1.78 ^r^	33.9 ^r^	2.75 ^r^	n.a.	n.a.	8	[88]
Cabernet Sauvignon (FRA)	4.49 ^r^	3.47 ^r^	72.2 ^r^	3.76 ^r^	n.a.	n.a.		
Chardonnay (FRA)	4.69 ^r^	5.90 ^r^	31.1 ^r^	2.70 ^r^	n.a.	n.a.		
Merlot (FRA)	4.61 ^r^	1.25 ^r^	29.6 ^r^	2.71 ^r^	n.a.	n.a.		
Pinot Noir (FRA)	4.38 ^r^	3.24 ^r^	57.6 ^r^	4.38 ^r^	n.a.	n.a.		
Prokupac (SRB)	4.67 ^r^	4.95 ^r^	15.8 ^r^	2.75 ^r^	n.a.	n.a.		
Riesling (GER)	4.13 ^r^	2.74 ^r^	54.6 ^r^	0.597 ^r^	n.a.	n.a.		
Sauvignon blanc (FRA	5.35 ^r^	4.05 ^r^	110 ^r^	2.75 ^r^	n.a.	n.a.		
Syrah (FRA)	3.58 ^r^	2.55 ^r^	0 ^r^	4.07 ^r^	n.a.	n.a.		
Vranac (MNE)	4.78 ^r^	3.11 ^r^	93.4 ^r^	0.986 ^r^	n.a.	n.a.		
Welschriesling (NN)	4.12 ^r^	3.08 ^r^	35.9 ^r^	2.73 ^r^	n.a.	n.a.		

* Country of origin: FRA—France, POR—Portugal, ESP—Spain, ITA—Italy, CRO—Croatia, MNE—Montenegro, GER—Germany, NN—unknown origin. ** Analysis method: 1—SLE, RP-HPLC, 2—SPE, HPLC, 3—LLE, HPLC, 4—SLE, HPLC, 5—SLE, SPE, UHPLC, 6—SLE, HPLC/Q-TOF MS/MS, 7—SPE, HPLC/QqTOF; *** n.a.—not analyzed. Results are expressed as follows: ^a^ mg/kg DW skin; ^b^ mg/kg; ^c^ mg/kg skins; ^d^ nmol/g grape tissue; ^e^ mg/kg FW skin; ^f^ μg/g fresh sample; ^g^ mg/kg berry, dry basis; ^h^ mg/g DM; ^i^ mg/kg frozen sample; ^j^ mg/100 g DM; ^k^ mg/100 g db; ^l^ mg/g FW; ^m^ mg/kg DM; ^n^ mg/100 g FM; ^o^ μg/100 g FW grapes; ^p^ μg/g; ^r^ mg/kg dry sample.

**Table 2:** Contents of hydroxycinnamic acids in the grapes and leaves of different grapevine cultivars.

Cultivar (Country of Origin *)	Caftaric Acid	Caffeic Acid	*trans*-Coutaric Acid	*trans*-Coumaric Acid	Fertaric Acid	Ferulic Acid	Analysis Method **	Ref.
	**Grapes–Red Cultivars**							
Aglianico (ITA)	320.4 ^d^	n.a. **	n.a.	n.a.	n.a.	n.a.	1	[77]
Alphonse Lavallee (FRA)	645.0 ^d^	n.a.	n.a.	n.a.	n.a.	n.a.	1	[77]
Azal Tinto (POR)	11 ^d^	n.a.	5.5 ^d^	n.a.	n.a.	n.a.	2	[90]
Borracal (ESP)	13 ^d^	n.a.	3.5 ^d^	n.a.	n.a.	n.a.	2	[90]
Brancelho (POR)	2.6 ^d^	n.a.	0.73 ^d^	n.a.	n.a.	n.a.	2	[90]
Cabernet Sauvignon (FRA)	n.a.	0.59 ^b^	n.a.	1.14 ^b^	n.a.	1.59 ^b^	3	[84]
	127.55 ^d^	Trace	84.86 ^d^	n.a.	3.62 ^d^	n.a.	4	[91]
	162.98 ^f^	50.57 ^f^	n.a.	12.76 ^f^	n.a.	0 ^f^	5	[79]
	54.9 ^l^	n.a.	2.3 ^l^	0 ^l^	n.a.	n.a.	6	[92]
	0 ^n^	901.2 ^n^	n.a.	367.5 ^n^	n.a.	412.3 ^n^	7	[78]
Cabernet Franc (FRA)	n.a.	0.50 ^b^	n.a.	0 ^b^	n.a.	10.33 ^b^	3	[84]
	0.1–2.6 ^i^	n.a.	n.a.	2.8–4.2 ^i^	n.a.	0.1–0.3 ^i^	5	[83]
Cesanese (ITA)	28.8 ^d^	n.a.	n.a.	n.a.	n.a.	n.a.	1	[77]
Docal (POR)	3.9 ^d^	n.a.	0.75 ^d^	n.a.	n.a.	n.a.	2	[90]
Espadeiro (POR)	44 ^d^	n.a.	8.9 ^d^	n.a.	n.a.	n.a.	2	[90]
Malvasia Nera (GRE)	171.9 ^d^	n.a.	n.a.	n.a.	n.a.	n.a.	1	[77]
Merlot (FRA)	n.a.	0.53 ^b^	n.a.	0 ^b^	n.a.	2.20 ^b^	3	[84]
	100.23 ^d^	Trace	39.00 ^d^	n.a.	2.72 ^d^	n.a.	4	[91]
	0 ^n^	1363.6 ^n^	n.a.	336.7 ^n^	n.a.	515.1 ^n^	7	[78]
	746.3 ^d^	n.a.	n.a.	n.a.	n.a.	n.a.	1	[77]
Nebbiolo (ITA)	0.05–0.08 ^f^	n.a.	0.05–0.53 ^f^	0.02–0.06 ^f^	n.a.	n.a.	1	[93]
Negroamaro (ITA)	8.5 ^d^	n.a.	n.a.	n.a.	n.a.	n.a.	1	[77]
Pedral (POR)	17 ^d^	n.a.	3.3 ^d^	n.a.	n.a.	n.a.	2	[90]
Pinot Noir (FRA)	n.a.	0.54 ^b^	n.a.	0 ^b^	n.a.	2.12 ^b^	3	[84]
	144–6195 ^e^	n.a. ^*^	14.8–1016 ^e^	n.a.	1.8–5.9 ^e^	n.a.	8	[94]
	53.9 ^k^	n.a.	n.a.	n.a.	n.a.	n.a.	5	[95]
Primitivo (CRO)	1.89 ^d^	n.a.	n.a.	n.a.	n.a.	n.a.	1	[77]
Sangiovese (ITA)	n.a.	0.84 ^b^	n.a.	5.59 ^b^	n.a.	6.62 ^b^	3	[84]
Susumaniello (ITA)	171.7 ^d^	n.a.	n.a.	n.a.	n.a.	n.a.	1	[77]
Syrah (FRA)	n.a.	0.67 ^b^	n.a.	4.39 ^b^	n.a.	6.98 ^b^	3	[84]
	n.a.	1.58 ^g^	n.a.	0 ^b^	n.a.	4.20 ^g^	5	[86]
	154.73 ^d^	Trace	136.05 ^d^	n.a.	2.62 ^d^	n.a.	9	[96]
Tempranillo (ESP)	0.8 ^f^	n.a.	0.9 ^f^	n.a.	0.65 ^f^	n.a.	2	[97]
Uva di Troia (ITA)	93.3 ^d^	n.a.	n.a.	n.a.	n.a.	n.a.	1	[77]
Vinhao (POR)	11 ^d^	n.a.	5.3 ^d^	n.a.	n.a.	n.a.	2	[90]
Vranac (MNE)	n.a. ^*^	0.085 ^a^	0.011 ^a^	0.009 ^a^	n.a.	n.a.	5	[98]
	**Grapes–White Cultivars**							
Albarino (ESP)	4.04 ^m^	n.a.	0.27 ^m^	1.96 ^m^	1.68 ^m^	n.a.	9	[87]
Chardonnay (FRA)	n.a.	0.58 ^b^	n.a.	0.72 ^b^	n.a.	13.95 ^b^	3	[84]
	1918 ^n^	1880.2 ^n^	n.a.	271.6 ^n^	n.a.	195.3 ^n^	7	[78]
Istrian Malvasia (CRO)	0.17–0.62 ^f^	n.a.	n.a.	n.a.	n.a.	n.a.	10	[99]
Moscato (NN)	48.4 ^d^	n.a.	n.a.	n.a.	n.a.	n.a.	1	[77]
Riesling (GER)	n.a.	0.65 ^b^	n.a.	0 ^b^	n.a.	11.83 ^b^	3	[84]
Sauvignon blanc (FRA)	n.a.	0.49 ^b^	n.a.	0 ^b^	n.a.	10.94 ^b^	3	[84]
	1430 ^n^	2498.4 ^n^	n.a.	275.1 ^n^	n.a.	136.4 ^n^	7	[78]
Semillon (FRA)	33.1 ^f^	n.a.	7.3 ^f^	n.a.	n.a.	n.a.	5	[100]
Viognier (FRA)	1075.2 ^n^	2285.1 ^n^	n.a.	301.7 ^n^	n.a.	140.7 ^n^	7	[78]
Vermentino (ITA)	1076.7 ^n^	1661.8 ^n^	n.a.	270.2 ^n^	n.a.	201.6 ^n^	7	[78]
Verdelho (POR)	3.2 ^d^	n.a.	1.3 ^d^	n.a.	n.a.	n.a.	2	[90]
Welschriesling (NN)	n.a.	0.63 ^b^	n.a.	0.22 ^b^	n.a.	8.95 ^b^	3	[84]
	**Leaves**							
Cabernet Franc (FRA)	n.a.	5.36 ^o^	n.a.	4.69 ^o^	n.a.	39.0 ^o^	11	[88]
Cabernet Sauvignon (FRA)	n.a.	5.21 ^o^	n.a.	0.772 ^o^	n.a.	22.4 ^o^		
Chardonnay (FRA)	n.a.	5.03 ^o^	n.a.	3.96 ^o^	n.a.	27.0 ^o^		
Merlot (FRA)	n.a.	2.72 ^o^	n.a.	2.84 ^o^	n.a.	18.3 ^o^		
Riesling (GER)	n.a.	4.18 ^o^	n.a.	1.29 ^o^	n.a.	19.1 ^o^		
Sangiovese (ITA)	n.a.	8.95 ^o^	n.a.	1.15 ^o^	n.a.	21.5 ^o^		
Sauvignon blanc (FRA)	n.a.	4.10 ^o^	n.a.	1.10 ^o^	n.a.	25.5 ^o^		
Syrah (FRA)	n.a.	4.96 ^o^	n.a.	1.91 ^o^	n.a.	18.2 ^o^		
Vranac (MNE)	n.a.	4.58 ^o^	n.a.	1.10 ^o^	n.a.	25.5 ^o^		
Welschriesling (NN)	n.a.	3.20 ^o^	n.a.	3.94 ^o^	n.a.	7.1 ^o^		

* Country of origin: FRA—France, POR—Portugal, ESP—Spain, ITA—Italy, CRO—Croatia, MNE—Montenegro, GER—Germany, NN—unknown origin. ** Analysis method: 1—SLE, RP-HPLC, 2—SPE, HPLC, 3—SLE, SPE, UHPLC, 4—SLE, HPLC-PDA-MS/MS, 5—SLE, HPLC, 6—SLE, LC-PDA-MS, 7—LLE, HPLC, 8—SLE, UHPLC, 9—SPE, HPLC/QqTOF, 10—SLE, HPLC/MS, 11—SLE, UHPLC-DAD MS/MS; *** n.a.—not analyzed. Results are expressed as follows: ^a^ mg/g DM; ^b^ mg/kg frozen sample; ^c^ mg/kg skin; ^d^ mg/kg DW ^e^ μg/g DM; ^f^ mg/kg; ^g^ mg/100 g db; ^h^ mg/kg FW skin; ^i^ μg/g fresh sample; ^j^ mg/; ^k^ mg/kg berry, dry basis; ^l^ mg/100 g DM; ^m^ mg/100 g FM; ^n^ μg/100 g FW grape; ^o^ mg/kg dry sample.

**Table 3:** Contents of stilbenes in grapes of different grapevine cultivars.

Cultivar (Country of Origin **)	*trans*-Resveratrol	*cis*-Resveratrol	*trans*-Piceid	*cis*-Piceid	Analysis Method *	Ref.
	**Grapes–Red Cultivars**					
Aglianico (ITA)	61.1 ^d^	n.a. ***	75.7 ^d^	n.a.	1	[77]
Alphonse Lavalleen (FRA)	40.0 ^d^	n.a.	24.1 ^d^	n.a.	1	[77]
Babić (CRO)	0.44 ^a^	0.42 ^a^	0.18 ^a^	n.a.	2	[108]
Brancelho (POR)	n.a.	n.a.	2.8 ^m^	n.a.	3	[90]
Borracal (ESP)	n.a.	n.a.	14 ^m^	n.a.	3	[90]
Cabernet Sauvignon (FRA)	0 ^e^	n.a.	69.77 ^e^	n.a.	2	[79]
	9.61 ^i^	n.a.	n.a.	n.a.	4	[84]
	56.4 ^n^	n.a.	n.a.	n.a.	5	[78]
	0.47 ^h^	n.a.	n.a.	n.a.	6	[91]
	n.a.	n.a.	5.2–6.9 ^h^	0 ^h^	7	[109]
	25.5 ^j^	n.a.	n.a.	n.a.	2	[39]
Gamay (FRA)	n.a.	n.a.	6.2–17.8 ^h^	0 ^h^	7	[109]
Merlot (FRA)	5.80 ^i^	n.a.	n.a.	n.a.	4	[84]
	1.02 ^a^	0.36 ^a^	0.31 ^a^	n.a.	2	[108]
	54.1 ^n^	n.a.	n.a.	n.a.	5	[78]
	9.2 ^d^	n.a.	26.3 ^d^	n.a.	1	[77]
	6.99 ^h^	n.a.	n.a.	n.a.	6	[91]
	n.a.	n.a.	25.6–28.1 ^h^	0–13.1 ^h^	7	[109]
	10.5 ^j^	n.a.	n.a.	n.a.	2	[39]
Negroamaro (ITA)	3.6 ^d^	n.a.	4.14 ^d^	n.a.	1	[77]
Pedral (POR)	n.a.	n.a.	38 ^m^	n.a.	3	[90]
Pinot Noir (FRA)	6.4–123 ^g^	n.a.	2.2–805 ^g^	n.a.	8	[94]
	5.80 ^i^	n.a.	n.a.	n.a.	4	[84]
	n.a.	n.a.	5.5 ^b^	n.a.	2	[95]
Pinot Gris (FRA)	n.a.	n.a.	2.1 ^b^	n.a.	2	[95]
Plavina (CRO)	0.30 ^a^	0.07 ^a^	0.17 ^a^	n.a.	2	[108]
Primitivo (CRO)	13.9 ^d^	n.a.	30.7 ^d^	n.a.	1	[77]
	1136.40 ^k^	n.a.	2332.10 ^k^	1776.20 ^k^	9	[105]
Prokupac (SRB)	13.42 ^i^	n.a.	n.a.	n.a.	4	[84]
Raboso Piave (ITA)	1134.80 ^k^	n.a.	395.30 ^k^	1476.80 ^k^	9	[105]
Syrah (FRA)	3.6 ^d^	n.a.	n.a.	n.a.	2	[86]
	0.08 ^h^	n.a.	n.a.	n.a.	6	[91]
Tempranillo (ESP)	0.12 ^e^	0.10 ^e^	0.65 ^e^	0.40 ^e^	3	[97]
Trnjak (CRO)	0.41 ^a^	1.74 ^a^	0.11 ^a^	n.a.	2	[108]
Vinhao (POR)	n.a.	n.a.	163 ^m^	n.a.	3	[90]
Vranac (MNE)	0.78 ^a^	0.93 ^a^	0 ^a^	n.a.	2	[108]
Verdelho (POR)	n.a.	n.a.	32 ^m^	n.a.	3	[90]
Albarino (ESP)	1.43 ^l^	n.a.	6.93 ^l^	n.a.	10	[87]
Chardonnay (FRA)	29.4 ^n^	n.a.	n.a.	n.a.	5	[78]
Istrian Malvasia (CRO)	n.a.	n.a.	0.01 ^f^	n.a.	11	[99]
Kujundžuša (CRO)	0.27 ^a^	0.84 ^a^	1.11 ^a^	n.a.	2	[108]
Zlatarica (CRO)	0.10 ^a^	0.26 ^a^	0.10 ^a^	n.a.	2	[108]
Maraština (CRO)	0.17 ^a^	0.29 ^a^	0.65 ^a^	n.a.	2	[108]
Debit (CRO)	0.72 ^a^	0.26 ^a^	0.29 ^a^	n.a.	2	[108]

* Country of origin: FRA—France, POR—Portugal, ESP—Spain, ITA—Italy, CRO—Croatia, MNE—Montenegro, GER—Germany, NN—unknown origin. ** Analysis method: 1—SLE, RP-HPLC, 2—SLE, HPLC, 3—SPE, HPLC, 4—SLE, SPE, UHPLC, 5—LLE, HPLC, 6—SLE, HPLC-PDA-MS/MS, 7—UAE, HPLC–MS, 8—SLE, UHPLC, 9—SLE, LC/QTOF, 10—SPE, HPLC/QqTOF, 11—SLE, HPLC/MS; *** n.a.—not analyzed; ** *V. labrusca* cultivars. Results are expressed as follows: ^a^ mg/kg FW grape berry; ^b^ mg/kg berry dry basis; ^c^ mg/kg FW skins; ^d^ mg/100 g db; ^e^ mg/kg; ^f^ mg/L; ^g^ μg/g DW; ^h^ mg/g DW; ^i^ mg/kg frozen sample; ^j^ mg/100 g DM; ^k^ μg/kg grape; ^l^ mg/100 g FM; ^m^ mg/kg DM; ^n^ μg/100 g FW.

**Table 4:** Contents of individual anthocyanins in the grapes of different red grapevine cultivars.

Cultivar (Country of Origin *)	3-*O*-Glucosides				3-*O*-Acetylglucosides				3-*O*-Coumaroylglucosides				3-*O*-Caffeoylglucosides					Analysis Method **	Ref.
	Dp ^1^	Cy ^2^	Pt ^3^	Pn ^4^	Mv ^5^	Dp	Cy	Pt	Pn	Mv	Dp	Cy	Pt	Pn	Mv	Pn	Mv		
Aglianico (ITA)	594.7 ^g^	52.1 ^g^	596.5 ^g^	553.6 ^g^	3348.0 ^g^	n.a.	n.a.	n.a.	n.a.	n.a.	n.a.	n.a.	n.a.	n.a.	n.a.	n.a.	n.a.	5	[77]
Alfrocheiro (POR)	0.03 ^k^	0 ^k^	0.16 ^k^	0.14 ^k^	2.90 ^k^	n.a.	0 ^k^	0 ^k^	0.04 ^k^	0.18 ^k^	n.a.	n.a.	0.06 ^k^	0.04 ^k^	1.46 ^k^	n.a.	n.a.	1	[121]
Alphonse Lavallee (FRA)	219.8 ^g^	46.4 ^g^	241.1 ^g^	52.5 ^g^	1851.1 ^g^	n.a.	n.a.	n.a.	n.a.	n.a.	n.a.	n.a.	n.a.	n.a.	n.a.	n.a.	n.a.	5	[77]
Alvarlhão (POR)	0.08 ^k^	0.24 ^k^	0.12 ^k^	1.04 ^k^	0.99 ^k^	n.a.	0.01 ^k^	0.01 ^k^	0.06 ^k^	0.05 ^k^	n.a.	n.a.	0.01 ^k^	0.12 ^k^	0.12 ^k^	n.a.	n.a.	1	[121]
Borracal (ESP)	481 ^b^	55 ^b^	513 ^b^	259 ^b^	1469 ^b^	n.a.	n.a.	n.a.	n.a.	n.a.	n.a.	n.a.	42 ^b^	26 ^b^	122 ^b^	n.a.	n.a.	7	[90]
Cabernet Sauvignon (FRA)	7124 ^c^	653 ^c^	2860 ^c^	560 ^c^	9119 ^c^	n.a. ***	n.a.	n.a.	n.a.	n.a.	n.a.	n.a.	n.a.	n.a.	n.a.	n.a.	n.a.	1	[79]
	1.59 ^g^	0.79 ^g^	7.40 ^g^	16.70 ^g^	328.86 ^g^	1.28 ^g^	0.17 ^g^	11.62 ^g^	32.08 ^g^	539.01 ^g^	0.37 ^g^	n.a.	2.52 ^g^	23.02 ^g^	71.08 ^g^	n.a.	0.16 ^g^	2	[91]
	2057 ^g^	43 ^g^	1558 ^g^	847 ^g^	6438 ^g^	502 ^g^	134 ^g^	408 ^g^	179 ^g^	1828 ^g^	376 ^g^	77 ^g^	315 ^g^	328 ^g^	2380 ^g^	39 ^g^	86 ^g^	3	[109]
	46 ^h^	Tr. ^h^	51 ^h^	43 ^h^	665 ^h^	21 ^h^	n.a.	26 ^h^	29 ^h^	425 ^h^	n.a.	Tr. ^h^	21 ^h^	n.a.	220 ^h^	n.a.	62 ^h^	1	[122]
	6540 ^g^	686 ^g^	1622 ^g^	1835 ^g^	9354 ^g^	687 ^g^	178 ^g^	542 ^g^	441 ^g^	4513 ^g^	178 ^g^	53 ^g^	130 ^g^	261 ^g^	1346 ^g^	43.63 ^g^	0 ^g^	4	[117]
	1206.86 ^g^	147.38 ^g^	719.03 ^g^	809.19 ^g^	5708.98 ^g^	384.02 ^g^	124.70 ^g^	342.41 ^g^	672.4 ^g^	4675.9 ^g^	124.26 ^g^	n.a.	n.a.	388.12 ^g^	1959.9 ^g^	n.a.	n.a.	3	[123]
	115.7 ^f^	53.8 ^f^	73.1 ^f^	106.2 ^f^	188.3 ^f^	31.5 ^f^	14.0 ^f^	23.0 ^f^	35.2 ^f^	55.4 ^f^	8.3 ^f^	5.1 ^f^	5.5 ^f^	17.4 ^f^	23.0 ^f^	1.6 ^f^	n.a.	1	[124]
Cabernet Franc (FRA)	0.95 ^k^	0.04 ^k^	1.03 ^k^	0.17 ^k^	4.10 ^k^	n.a.	0.01 ^k^	0.02 ^k^	0.20 ^k^	0.27 ^k^	n.a.	n.a.	0.22 ^k^	0.02 ^k^	1.52 ^k^	n.a.	n.a.	1	[121]
Carignan Noir (FRA)	0.34 ^k^	0.02 ^k^	0.57 ^k^	0.56 ^k^	5.60 ^k^	n.a.	0 ^k^	0 ^k^	0.04 ^k^	0.53 ^k^	n.a.	n.a.	0.16 ^k^	0.35 ^k^	3.44 ^k^	n.a.	n.a.	1	[121]
Gamay (FRA)	200 ^g^	57 ^g^	259 ^g^	534 ^g^	3847 ^g^	0 ^g^	0 ^g^	0 ^g^	0 ^g^	131 ^g^	0 ^g^	0 ^g^	0 ^g^	37 ^g^	189 ^g^	0 ^g^	0 ^g^	3	[109]
	0.02 ^k^	0 ^k^	0.08 ^k^	0.12 ^k^	1.77 ^k^	n.a.	0 ^k^	0 ^k^	0.04 ^k^	0.23 ^k^	n.a.	n.a.	0.02 ^k^	0.11 ^k^	1.61 ^k^	n.a.	n.a.	1	[121]
Gewürztraminer (NN)	0.21 ^k^	0.01 ^k^	0.44 ^k^	0.26 ^k^	4.92 ^k^	n.a.	0.03 ^k^	0.39 ^k^	0.07 ^k^	1.54 ^k^	n.a.	n.a.	0.39 ^k^	0.07 ^k^	0.88 ^k^	n.a.	n.a.	1	[121]
Grenache (ESP)	n.a.	42.9 ^c^	98.8 ^c^	156 ^c^	355 ^c^	n.a.	n.a.	n.a.	n.a.	n.a.	n.a.	n.a.	n.a.	n.a.	n.a.	n.a.	n.a.	1	[125]
	0.25 ^k^	0.04 ^k^	0.14 ^k^	0.18 ^k^	1.44 ^k^	n.a.	0 ^k^	0 ^k^	0 ^k^	0.01 ^k^	n.a.	n.a.	0.05 ^k^	0.05 ^k^	0.17 ^k^	n.a.	n.a.	1	[121]
Merlot (FRA)	6.91 ^g^	1.78 ^g^	25.05 ^g^	58.89 ^g^	251.54 ^g^	3.39 ^g^	0.65 ^g^	19.92 ^g^	47.79 ^g^	258.20 ^g^	3.57 ^g^	n.a.	7.04 ^g^	23.02 ^g^	49.58 ^g^	n.a.	0.47 ^g^	2	[91]
	2059 ^g^	363 ^g^	1470 ^g^	295 ^g^	6835 ^g^	450 ^g^	133 ^g^	350 ^g^	286 ^g^	1813 ^g^	201 ^g^	254 ^g^	169 ^g^	218 ^g^	1202 ^g^	0 ^g^	0 ^g^	3	[109]
	974.5 ^g^	25.93 ^g^	534.4 ^g^	580.2 ^g^	12180 ^g^	n.a.	n.a.	n.a.	n.a.	n.a.	n.a.	n.a.	n.a.	n.a.	n.a.	n.a.	n.a.	5	[77]
	1544.63 ^g^	310.21 ^g^	1252.03 ^g^	1357.51 ^g^	8027.00 ^g^	495.00 ^g^	161.14 ^g^	340.34 ^g^	168.16 ^g^	4092.97 ^g^	228.73 ^g^	n.a.	n.a.	369.91 ^g^	2415.44 ^g^	n.a.	n.a.	3	[123]
	250.6 ^f^	87.5 ^f^	125.6 ^f^	74.2 ^f^	203.0 ^f^	46.5 ^f^	15.4 ^f^	29.2 ^f^	12.7 ^f^	43.6 ^f^	16.6 ^f^	9.5 ^f^	9.4 ^f^	8.0 ^f^	20.6 ^f^	2.1 ^f^	n.a.	1	[124]
Moristel (ESP)	n.a.	12.1 ^c^	56.8 ^c^	33.7 ^c^	265 ^c^	n.a.	n.a.	n.a.	n.a.	n.a.	n.a.	n.a.	n.a.	n.a.	n.a.	n.a.	n.a.	1	[125]
Negroamaro (ITA)	112.8 ^g^	50.6 ^g^	203.6 ^g^	110.2 ^g^	662.6 ^g^	n.a.	n.a.	n.a.	n.a.	n.a.	n.a.	n.a.	n.a.	n.a.	n.a.	n.a.	n.a.	5	[77]
Pinot Noir (FRA)	2.9 ^b^	4.4 ^b^	6.4 ^b^	34.9 ^b^	61.5 ^b^	n.a.	n.a.	n.a.	n.a.	n.a.	n.a.	n.a.	n.a.	n.a.	n.a.	n.a.	n.a.	6	[94]
	2373 ^g^	1325 ^g^	1249 ^g^	1528 ^g^	6019 ^g^	337 ^g^	90.2 ^g^	274 ^g^	288 ^g^	901 ^g^	0 ^g^	0 ^g^	142 ^g^	88 ^g^	597 ^g^	0 ^g^	0 ^g^	3	[109]
	105.6 ^f^	24.1 ^f^	104.5 ^f^	114.2 ^f^	378.5 ^f^	0 ^f^	0 ^f^	0 ^f^	0 ^f^	0 ^f^	0 ^f^	0 ^f^	0 ^f^	0 ^f^	0 ^f^	0 ^f^	n.a.	1	[124]
Primitivo (CRO)	44.7 ^g^	14.7 ^g^	168.4 ^g^	207.7 ^g^	1883.0 ^g^	n.a.	n.a.	n.a.	n.a.	n.a.	n.a.	n.a.	n.a.	n.a.	n.a.	n.a.	n.a.	5	[77]
Syrah (FRA)	3.30 ^g^	0.70 ^g^	24.08 ^g^	48.42 ^g^	308.45 ^g^	1.86 ^g^	0.18 ^g^	17.68 ^g^	72.53 ^g^	816.78 ^g^	8 ^g^	n.a.	17.20 ^g^	63.86 ^g^	251.70 ^g^	n.a.	2.46 ^g^	2	[91]
	1660 ^g^	111 ^g^	987 ^g^	679 ^g^	3574 ^g^	164 ^g^	0 ^g^	231 ^g^	142 ^g^	1303 ^g^	0 ^g^	0 ^g^	0 ^g^	288 ^g^	1629 ^g^	0 ^g^	0 ^g^	3	[109]
Tempranillo (ESP)	n.a.	17.5 ^c^	76.6 ^c^	21.8 ^c^	239 ^c^	n.a.	n.a.	n.a.	n.a.	n.a.	n.a.	n.a.	n.a.	n.a.	n.a.	n.a.	n.a.	1	[125]
	275.68 ^f^	107.81 ^f^	204.33 ^f^	207.57 ^f^	608.36 ^f^	7.02 ^f^	1.09 ^f^	4.86 ^f^	2.38 ^f^	20.07 ^f^	36.73 ^f^	16.21 ^f^	29.68 ^f^	24.66 ^f^	205.25 ^f^	0.34 ^f^	2.54 ^f^	1	[126]
	256.94 ^c^	84.28 ^c^	164.80 ^c^	115.37 ^c^	342.44 ^c^	4.49 ^c^	0.83 ^c^	2.85 ^c^	0.20 ^c^	5.82 ^c^	24.37 ^c^	7.99 ^c^	15.49 ^c^	11.85 ^c^	42.06 ^c^	n.a.	0.48 ^c^	7	[97]
	107 ^h^	15 ^h^	91 ^h^	30 ^h^	292 ^h^	10 ^h^	n.a.	15 ^h^	Tr. ^h^	49 ^h^	n.a.	Tr. ^h^	56 ^h^	n.a.	220 ^h^	n.a.	Tr. ^h^	1	[122]
Tinta Barroca (POR)	0.21 ^k^	0.06 ^k^	0.41 ^k^	0.57 ^k^	4.93 ^k^	n.a.	0 ^k^	0 ^k^	0.07 ^k^	0.26 ^k^	n.a.	n.a.	0.11 ^k^	0.13 ^k^	1.94 ^k^	n.a.	n.a.	1	[121]
Tinto Cão (POR)	0.27 ^k^	0.01 ^k^	0.39 ^k^	0.13 ^k^	2.65 ^k^	n.a.	0 ^k^	0.01 ^k^	0.16 ^k^	0.37 ^k^	n.a.	n.a.	0.25 ^k^	0.05 ^k^	2.57 ^k^	n.a.	n.a.	1	[121]
Touriga Francesca (POR)	0.05 ^i^	0.01 ^i^	0.08 ^i^	0.02 ^i^	1.02 ^i^	0 ^i^	0.01 ^i^	0.06 ^i^	0.31 ^i^	0.11 ^i^	0 ^i^	0.01 ^i^	0.11 ^i^	0.08 ^i^	1.25 ^i^	0.01 ^i^	0.10 ^i^	1	[127]
Touriga Nacional (POR)	691.2 ^a^	0.73 ^a^	1761 ^a^	542 ^a^	2232 ^a^	n.a.	n.a.	n.a.	n.a.	n.a.	n.a.	n.a.	n.a.	n.a.	n.a.	n.a.	n.a.	8	[68]
	0.48 ^i^	0.07 ^i^	0.47 ^i^	0.52 ^i^	2.51 ^i^	0.05 ^i^	0.01 ^i^	0.07 ^i^	0.09 ^i^	0.52 ^i^	0.01 ^i^	0 ^i^	0.13 ^i^	0.22 ^i^	0.85 ^i^	0.01 ^i^	0.12 ^i^	1	[127]
Verdelho tinto (POR)	318 ^b^	28 ^b^	320 ^b^	145 ^b^	1486 ^b^	n.a.	n.a.	n.a.	n.a.	n.a.	n.a.	n.a.	56 ^b^	32 ^b^	260 ^b^	n.a.	n.a.	7	[90]
Vinhao (POR)	3653 ^b^	409 ^b^	2054 ^b^	623 ^b^	4736 ^b^	n.a.	n.a.	n.a.	n.a.	n.a.	n.a.	n.a.	106 ^b^	29 ^b^	233 ^b^	n.a.	n.a.	7	[90]
	745 ^g^	243 ^g^	735 ^g^	706 ^g^	3579 ^g^	45 ^g^	0 ^g^	44 ^g^	103 ^g^	172 ^g^	0 ^g^	0 ^g^	102 ^g^	114 ^g^	754 ^g^	0 ^g^	0 ^g^	3	[109]
Vranac (MNE)	3.28 ^d^	0.98 ^d^	3.60 ^d^	2.41 ^d^	7.09 ^d^	n.a.	n.a.	n.a.	n.a.	n.a.	n.a.	n.a.	n.a.	n.a.	n.a.	n.a.	n.a.	1	[98]

* Country of origin: FRA—France, POR—Portugal, ESP—Spain, ITA—Italy, CRO—Croatia, MNE—Montenegro, NN—unknown origin. ** Analysis method: 1—SLE, RP-HPLC, 2—SLE, HPLC, 3—SPE, HPLC, 4—SLE, SPE, UHPLC, 5—LLE, HPLC, 6—SLE, HPLC-PDA-MS/MS, 7—UAE, HPLC–MS, 8—SLE, UHPLC, 9—SLE, LC/QTOF, 10—SPE, HPLC/QqTOF, 11—SLE, HPLC/MS; *** n.a.—not analyzed. Results are expressed as follows: ^a^ μg/g; ^b^ μg/g DW; ^c^ mg/kg; ^d^ mg/g DM; ^e^ mg/kg berry, dry basis; ^f^ mg/kg grape; ^g^ mg/kg DW ^h^ mg/kg FW berry; ^i^ mg/g berry; ^j^ mg/100 g DM; ^k^ mg/g skin; ^1^ Delphinidin; ^2^ Cyanidin; ^3^ Petunidin; ^4^ Peonidin; ^5^ Malvidin.

**Table 5:** Flavonol profiles of different grapevine cultivars.

Cultivar (Country of Origin *)	Myricetin			Quercetin			Kaempferol			Isorhamnetin			Syringentin	Laricitrin	Analysis Method **	Ref.
	Glc ^1^	Glr ^1^	Gal ^3^	Glc	Glr	Rut ^4^	Gal	Glc	Glr	Gal	Glc	Glr	Glc	Glc		
	**Grapes–Red Cultivars**															
Alphonse Lavallee (FRA)	n.a. ***	n.a.	n.a.	4.1 ^d^	n.a.	0 ^d^	n.a.	4.3 ^d^	n.a.	n.a.	n.a.	n.a.	n.a.	n.a.	1	[77]
Aglianico (ITA)	n.a.	n.a.	n.a.	6.7 ^d^	n.a.	6.1 ^d^	n.a.	20.9 ^d^	n.a.	n.a.	n.a.	n.a.	n.a.	n.a.	1	[77]
Azal Tinto (POR)	21 ^d^	n.a.	n.a.	27 ^d^	n.a.	n.a.	n.a.	2.9 ^d^	n.a.	n.a.	26 ^d^	n.a.	21 ^d^	15 ^d^	2	[90]
Babić (CRO)	n.a.	n.a.	n.a.	1.18 ^c^	n.a.	n.a.	n.a.	n.a.	n.a.	n.a.	n.a.	n.a.	n.a.	n.a.	2	[108]
Borracal (POR)	32 ^d^	n.a.	n.a.	40 ^d^	n.a.	n.a.	n.a.	4.4 ^d^	n.a.	n.a.	22 ^d^	n.a.	26 ^d^	11 ^d^	2	[90]
Brancelho (POR)	8.4 ^d^	n.a.	n.a.	30 ^d^	n.a.	n.a.	n.a.	5.1 ^d^	n.a.	n.a.	21 ^d^	n.a.	15 ^d^	8.0 ^d^	2	[90]
Cabernet Sauvignon (FRA)	279.87 ^d^	n.a.	n.a.	754.37 ^d^	n.a.	35.36 ^d^	n.a.	n.a.	n.a.	n.a.	73.91 ^d^	n.a.	n.a.	n.a.	2	[79]
	198.92 ^d^	3.96 ^d^	n.a.	n.a.	46.79 ^d^	n.a.	n.a.	0.52 ^d^	n.a.	n.a.	34.46 ^d^	n.a.	29.80 ^d^	5.56 ^d^	3	[91]
	95.4 ^d^	n.a.	n.a.	76.7 ^d^	61.3 ^d^	n.a.	16.7 ^d^	71.4 ^d^	n.a.	10.5 ^d^	91.7 ^d^	0 ^d^	136.3 ^d^	22.9 ^d^	4	[109]
	22 ^c^	10 ^c^	n.a.	48 ^c^	59 ^c^	n.a.	n.a.	13 ^c^	n.a.	n.a.	28 ^c^	n.a.	n.a.	n.a.	2	[134]
	248.86 ^d^	n.a.	n.a.	297.33 ^d^	159.52 ^d^	n.a.	59.68 ^d^	197.36 ^d^	n.a.	39.51 ^d^	261.25 ^d^	0 ^d^	140.88 ^d^	115.61 ^d^	5	[114]
	168.08 ^d^	n.a.	417.60 ^d^	23.55 ^d^	120.88 ^d^	150.90 ^d^	46.50 ^d^	0 ^d^	n.a.	148.10 ^d^	n.a.	n.a.	109.38 ^d^	30.15 ^d^	4	[123]
Cesanese (ITA)	n.a.	n.a.	n.a.	2.87 ^d^	n.a.	54.7 ^d^	n.a.	40.7 ^d^	n.a.	n.a.	n.a.	n.a.	n.a.	n.a.	1	[77]
Docal (POR)	41 ^d^	n.a.	n.a.	25 ^d^	n.a.	n.a.	n.a.	3.4 ^d^	n.a.	n.a.	15 ^d^	n.a.	27 ^d^	20 ^d^	6	[90]
Espadeiro (POR)	33 ^d^	n.a.	n.a.	93 ^d^	n.a.	n.a.	n.a.	23 ^d^	n.a.	n.a.	48 ^d^	n.a.	38 ^d^	45 ^d^	6	[90]
Gamay (FRA)	0 ^d^	n.a.	n.a.	10.3 ^d^	30.7 ^d^	n.a.	0 ^d^	0 ^d^	n.a.	12.4 ^d^	46.7 ^d^	0 ^d^	46.1 ^d^	9.5 ^d^	4	[109]
Gewürtztramminer (NN)	0 ^c^	0 ^c^	n.a.	24 ^c^	17 ^c^	n.a.	n.a.	6.7 ^c^	n.a.	n.a.	2.3 ^c^	n.a.	n.a.	n.a.	2	[134]
Malvasia Nera (ITA)	n.a.	n.a.	n.a.	9.0 ^d^	n.a.	0 ^d^	n.a.	31.2 ^d^	n.a.	n.a.	n.a.	n.a.	n.a.	n.a.	1	[77]
Merlot (FRA)	n.a.	n.a.	n.a.	1.65 ^c^	n.a.	n.a.	n.a.	n.a.	n.a.	n.a.	n.a.	n.a.	n.a.	n.a.	2	[108]
	75.92 ^d^	1.57 ^d^	n.a.	n.a.	35.18 ^d^	n.a.	n.a.	0.20 ^d^	n.a.	n.a.	11.41 ^d^	n.a.	7.16 ^d^	2.21 ^d^	3	[91]
	n.a.	n.a.	n.a.	45.0 ^d^	n.a.	30.2 ^d^	n.a.	97.7 ^d^	n.a.	n.a.	n.a.	n.a.	n.a.	n.a.	1	[77]
	150 ^d^	n.a.	n.a.	67.4 ^d^	58.9 ^d^	n.a.	0 ^d^	65.9 ^d^	n.a.	0 ^d^	134.8 ^d^	0 ^d^	82.2 ^d^	28.3 ^d^	4	[109]
	142.22 ^d^	n.a.	286.82 ^d^	150.00 ^d^	34.80 ^d^	144.54 ^d^	39.52 ^d^	0 ^d^	n.a.	32.47 ^d^	n.a.	n.a.	103.46 ^d^	29.24 ^d^	4	[123]
	13 ^c^	5.8 ^c^	n.a.	31 ^c^	43 ^c^	n.a.	n.a.	8 ^c^	n.a.	n.a.	17 ^c^	n.a.	n.a.	n.a.	2	[134]
Negroamaro (ITA)	n.a.	n.a.	n.a.	1.03 ^d^	n.a.	1.39 ^d^	n.a.	32.0 ^d^	n.a.	n.a.	n.a.	n.a.	n.a.	n.a.	1	[77]
Padeiro de Basto (POR)	28 ^d^	n.a.	n.a.	15 ^d^	n.a.	n.a.	n.a.	23 ^d^	n.a.	n.a.	17 ^d^	n.a.	61 ^d^	22 ^d^	6	[90]
Pedral (POR)	11 ^d^	n.a.	n.a.	13 ^d^	n.a.	n.a.	n.a.	3.3 ^d^	n.a.	n.a.	4.7 ^d^	n.a.	15 ^d^	6.2 ^d^	6	[90]
Pinot Noir (FRA)	20.3 ^i^	n.a.	n.a.	67.2 ^i^	n.a.	30.5 ^i^	33.8 ^i^	25.8 ^i^	n.a.	n.a.	13.0 ^i^	n.a.	8.6 ^i^	n.a.	1	[95]
	1066 ^b^	391 ^b^	n.a.	105 ^b^	2726 ^b^	272 ^b^	240 ^b^	177 ^b^	n.a.	n.a.	319 ^b^	n.a.	171 ^b^	n.a.	7	[94]
	88.1 ^d^	n.a.	n.a.	28.5 ^d^	31.7 ^d^	n.a.	0 ^d^	0 ^d^	n.a.	0 ^d^	0 ^d^	0 ^d^	35.5 ^d^	16.9 ^d^	4	[109]
Plavina (CRO)	n.a.	n.a.	n.a.	0.79 ^c^	n.a.	n.a.	n.a.	n.a.	n.a.	n.a.	n.a.	n.a.	n.a.	n.a.	2	[108]
Primitivo (CRO)	38.1 ^d^	n.a.	n.a.	11.5 ^d^	53.5 ^d^	n.a.	0 ^d^	27.7 ^d^	n.a.	0 ^d^	0 ^d^	0 ^d^	166.3 ^d^	0 ^d^	2	[108]
	n.a.	n.a.	n.a.	6.3 ^d^	n.a.	6.2 ^d^	n.a.	33.9 ^d^	n.a.	n.a.	n.a.	n.a.	n.a.	n.a.	1	[77]
Susumaniello (ITA)	n.a.	n.a.	n.a.	7.6 ^d^	n.a.	10.6 ^d^	n.a.	22.0 ^d^	n.a.	n.a.	n.a.	n.a.	n.a.	n.a.	1	[77]
Syrah (FRA)	163.70 ^f^	n.a.	15.04 ^f^	675.34 ^f^	n.a.	n.a.	93.8 ^f^	116.05 ^f^	4.83 ^f^	293.67 ^f^	181.44 ^f^	n.a.	n.a.	n.a.	2	[80]
	209.61 ^d^	1.06 ^d^	n.a.	n.a.	10.95 ^d^	n.a.	n.a.	0.14 ^d^	n.a.	n.a.	50.24 ^d^	n.a.	25.38 ^d^	4.32 ^d^	3	[91]
	181.8 ^d^	n.a.	n.a.	358.6 ^d^	155.7 ^d^	n.a.	56.2 ^d^	118.3 ^d^	n.a.	34.3 ^d^	265.6 ^d^	60.8 ^d^	107.9 ^d^	35 ^d^	4	[109]
	21 ^c^	7.3 ^c^	n.a.	55 ^c^	35 ^c^	n.a.	n.a.	18 ^c^	n.a.	n.a.	48 ^c^	n.a.	n.a.	n.a.	2	[134]
	260.90 ^d^	n.a.	n.a.	1411.45 ^d^	210.96 ^d^	n.a.	213.86 ^d^	378.97 ^d^	n.a.	91.68 ^d^	937.34 ^d^	255.79 ^d^	305.59 ^d^	175.53 ^d^	5	[66]
Tempranillo (SPA)	1.59 ^e^	18.76 ^e^	0.72 ^e^	6.20 ^e^	4.60 ^e^	0.39 ^e^	0.57 ^e^	0.77 ^e^	0.16 ^e^	0.18 ^e^	0.40 ^e^	0.11 ^e^	0.71 ^e^	2.30 ^e^	4	[97]
Trnjak (CRO)	n.a.	n.a.	n.a.	1.16 ^c^	n.a.	n.a.	n.a.	n.a.	n.a.	n.a.	n.a.	n.a.	n.a.	n.a.	2	[108]
Uva di Troia (ITA)	n.a.	n.a.	n.a.	15.0 ^d^	n.a.	41.4 ^d^	n.a.	45.8 ^d^	n.a.	n.a.	n.a.	n.a.	n.a.	n.a.	1	[77]
Vinhao (POR)	163 ^d^	n.a.	n.a.	13 ^d^	n.a.	n.a.	n.a.	1.6 ^d^	n.a.	n.a.	7.6 ^d^	n.a.	26 ^d^	21 ^d^	6	[90]
Verdelho tinto (POR)	56 ^d^	n.a.	n.a.	58 ^d^	n.a.	n.a.	n.a.	11 ^d^	n.a.	n.a.	13 ^d^	n.a.	31 ^d^	22 ^d^	6	[90]
Vranac (MNE)	0.11 ^h^	n.a.	n.a.	0.32 ^h^	n.a.	0.44 ^h^	n.a.	0.09 ^h^	n.a.	n.a.	n.a.	n.a.	n.a.	n.a.	2	[98]
	n.a.	n.a.	n.a.	1.73 ^c^	n.a.	n.a.	n.a.	n.a.	n.a.	n.a.	n.a.	n.a.	n.a.	n.a.	2	[108]
	**Grapes–White Cultivars**															
Albarino (POR)	n.a.	n.a.	n.a.	12.43 ^j^	0.98 ^j^	0.42 ^j^	n.a.	8.43 ^j^	3.21 ^j^	n.a.	n.a.	n.a.	n.a.	n.a.	8	[87]
Chardonnay (FRA)	0 ^c^	0 ^c^	n.a.	17 ^c^	25 ^c^	n.a.	n.a.	8.4 ^c^	n.a.	n.a.	0.48 ^c^	n.a.	n.a.	n.a.	2	[134]
	0 ^d^	n.a.	n.a.	587.49 ^d^	174.60 ^d^	n.a.	124.55 ^d^	277.05 ^d^	n.a.	0 ^d^	0 ^d^	0 ^d^	0 ^d^	60.25 ^d^	5	[66]
	0 ^d^	n.a.	0 ^d^	161.66 ^d^	67.34 ^d^	78.85 ^d^	6.64 ^d^	15.20 ^d^	n.a.	44.90 ^d^	n.a.	n.a.	0 ^d^	0 ^d^	4	[123]
Debit (CRO)	n.a.	n.a.	n.a.	0.49 ^c^	n.a.	n.a.	n.a.	n.a.	n.a.	n.a.	n.a.	n.a.	n.a.	n.a.	2	[108]
Istrian Malvasia (CRO)	n.a.	n.a.	n.a.	178.6 ^g^	12.3 ^g^	6.7 ^g^	52.3 ^g^	62.2 ^g^	1.1 ^g^	12.7 ^g^	n.a.	n.a.	n.a.	n.a.	9	[99]
Italian Riesling (NN)	0 ^d^	n.a.	n.a.	74.29 ^d^	128.63 ^d^	n.a.	13.75 ^d^	36.46 ^d^	n.a.	0 ^d^	0 ^d^	0 ^d^	0 ^d^	0 ^d^	5	[66]
	0 ^d^	n.a.	0 ^d^	35.29 ^d^	106.27 ^d^	164.56 ^d^	72.56 ^d^	5.96 ^d^	n.a.	38.89 ^d^	n.a.	n.a.	0 ^d^	0 ^d^	4	[123]
Kujundžuša (CRO)	n.a.	n.a.	n.a.	2.55 ^c^	n.a.	n.a.	n.a.	n.a.	n.a.	n.a.	n.a.	n.a.	n.a.	n.a.	2	[108]
Maraština (CRO)	n.a.	n.a.	n.a.	0.21 ^c^	n.a.	n.a.	n.a.	n.a.	n.a.	n.a.	n.a.	n.a.	n.a.	n.a.	2	[108]
Moscato (ITA)	n.a.	n.a.	n.a.	136.5 ^d^	n.a.	25.2 ^d^	n.a.	101.0 ^d^	n.a.	n.a.	n.a.	n.a.	n.a.	n.a.	1	[77]
Rabo de Ovelha (POR)	40 ^d^	n.a.	n.a.	26 ^d^	n.a.	n.a.	n.a.	31 ^d^	n.a.	n.a.	15 ^d^	n.a.	28 ^d^	19 ^d^	6	[90]
Riesling (GER)	0 ^c^	0 ^c^	n.a.	22 ^c^	30 ^c^	n.a.	n.a.	2.9 ^c^	n.a.	n.a.	Tr. ^c^	n.a.	n.a.	n.a.	2	[134]
Sauvignon Blanc (FRA)	0 ^c^	0 ^c^	n.a.	8.9 ^c^	12 ^c^	n.a.	n.a.	2.0 ^c^	n.a.	n.a.	0.78 ^c^	n.a.	n.a.	n.a.	2	[134]
Zlatarica (CRO)	n.a.	n.a.	n.a.	0.58 ^c^	n.a.	n.a.	n.a.	n.a.	n.a.	n.a.	n.a.	n.a.	n.a.	n.a.	2	[108]

* Country of origin: FRA—France, POR—Portugal, ESP—Spain, ITA—Italy, CRO—Croatia, MNE—Montenegro, NN—unknown origin. ** Analysis method: 1—SLE, RP-HPLC, 2—SLE, HPLC, 3—SLE, HPLC-PDA-MS/MS, 4—UAE, HPLC—MS, 5—SLE, HPLC–MSD, 6—SPE, HPLC, 7—SLE, UHPLC, 8—SPE, HPLC/QqTOF, 9—SLE, HPLC/MS; *** n.a.—not analyzed. Results are expressed as follows: ^a^ mg/kg FW skin; ^b^ μg/g DW; ^c^ mg/kg FW grape berry; ^d^ mg/kg DW; ^e^ mg/kg; ^f^ mg/kg skins; ^g^ mg/L; ^h^ mg/g DM; ^i^ mg/kg berry, dry basis; ^j^ mg/100 g FW; ^1^ Glucoside; ^2^ Glucuronide; ^3^ Rutinoside; ^4^ Galactoside.

**Table 6:** Contents of flavan-3-ols in grapes of different grapevine cultivars.

Cultivar	Catechin	Epicatechin	Galocatechin	Epigallocatechin	Epicatechin Gallate	Epigallocatechin Gallate	Procyanidins				Analysis Method	Ref.
							B1	B2	B3	B4		
**Grapes–Red Cultivars**												
Aglianico (ITA)	3214.9 ^a^	1890.1 ^a^	n.a.	n.a.	n.a.	n.a.	n.a.	n.a.	n.a.	n.a.	1	[77]
Alphonse Lavallee (FRA)	331.20 ^a^	32.4 ^a^	n.a.	n.a.	n.a.	n.a.	n.a.	n.a.	n.a.	n.a.	1	[99]
Babić (CRO)	2.6 ^c^	0 ^c^	n.a.	n.a.	0.45 ^c^	n.a.	1.53 ^c^	1.19 ^c^	n.a.	n.a.	2	[108]
Cabernet Franc (FRA)	0 ^n^	0 ^n^	3.81 ^n^	2.19 ^n^	n.a.	0 ^n^	n.a.	n.a.	n.a.	n.a.	3	[84]
Cabernet Sauvignon (FRA)	102.4 ^b^	28.79 ^b^	n.a.	82.71 ^b^	n.a.	n.a.	39.17 ^b^	23.83 ^b^	n.a.	n.a.	2	[79]
	5.9 ^n^	3.01 ^n^	4.13 ^n^	1.95 ^n^	n.a.	0 ^n^	n.a.	n.a.	n.a.	n.a.	3	[84]
	73.5 ^r^	792.4 ^r^	n.a. ^*^	n.a.	n.a.	n.a.	n.a.	n.a.	n.a.	n.a.	4	[78]
	28.37 ^a^	57.45 ^a^	n.a.	n.a.	6.81 ^a^	n.a.	n.a.	n.a.	n.a.	n.a.	5	[91]
	23.5 ^a^	0 ^a^	n.a.	n.a.	n.a.	n.a.	n.a.	n.a.	n.a.	n.a.	6	[109]
	4.4 ^c^	0.4 ^c^	n.a.	n.a.	n.a.	n.a.	n.a.	n.a.	n.a.	n.a.	2	[122]
	17 ^k^	6.2 ^k^	n.a.	n.a.	n.a.	n.a.	12 ^k^	0.99 ^k^	27 ^k^	n.a.	2	[134]
	0.31 ^h^	0.1 ^h^	n.a.	n.a.	0.08 ^h^	n.a.	0.11	0.84 ^h^	0.25 ^h^	0 ^h^	2	[138]
	118.42 ^f^	32.38 ^f^	n.a.	n.a.	n.a.	1.96 ^f^	n.a.	n.a.	n.a.	n.a.	12	[140]
	2.6 ^l^	0.9 ^l^	1.8 ^l^	1.5 ^l^	n.a.	n.a.	n.a.	n.a.	n.a.	n.a.	7	[141]
	0.206 ^m^	0.171 ^m^	n.a.	n.a.	0 ^m^	n.a.	0 ^m^	0 ^m^	0 ^m^	0 ^m^	8	[142]
	0.212 ^h^	0.119 ^h^	n.a.	n.a.	0 ^h^	n.a.	0.005 ^h^	0 ^h^	0.04 ^h^	0 ^h^	9	[143]
Carmenere (FRA)	3.5 ^l^	1.6 ^l^	2.8 ^l^	2.5 ^l^	n.a.	n.a.	n.a.	n.a.	n.a.	n.a.	7	[141]
Cesanese (ITA)	178.8 ^a^	8.9 ^a^	n.a.	n.a.	n.a.	n.a.	n.a.	n.a.	n.a.	n.a.	1	[77]
Gamay (FRA)	19.2 ^a^	0 ^a^	n.a.	n.a.	n.a.	n.a.	n.a.	n.a.	n.a.	n.a.	6	[109]
Gewurztraminer (NN)	19 ^k^	8.3 ^k^	n.a.	n.a.	n.a.	n.a.	21 ^k^	48 ^k^	n.a.	n.a.	2	[134]
Graciano (SPA)	0.104 ^m^	0.235 ^m^	n.a.	n.a.	0 ^m^	n.a.	0 ^m^	0 ^m^	0 ^m^	0 ^m^	8	[142]
Jean Tinto (FRA)	3.2 ^c^	0.6 ^c^	n.a.	n.a.	n.a.	n.a.	n.a.	n.a.	n.a.	n.a.	2	[122]
Malvasia Nera (ITA)	966.0 ^a^	734.7 ^a^	n.a.	n.a.	n.a.	n.a.	n.a.	n.a.	n.a.	n.a.	1	[77]
Merlot (FRA)	7.47 ^n^	0 ^n^	4.62 ^n^	2.24 ^n^	n.a.	0 ^n^	n.a.	n.a.	n.a.	n.a.	3	[84]
	16 ^o^	13 ^o^	n.a.	n.a.	n.a.	n.a.	n.a.	n.a.	n.a.	n.a.	2	[85]
	74.2 ^r^	389.2 ^r^	n.a.	n.a.	n.a.	n.a.	n.a.	n.a.	n.a.	n.a.	4	[78]
	601.0 ^a^	980.7 ^a^	n.a.	n.a.	n.a.	n.a.	n.a.	n.a.	n.a.	n.a.	1	[77]
	20.85 ^a^	68.09 ^a^	n.a.	n.a.	75.3 ^a^	n.a.	n.a.	n.a.	n.a.	n.a.	5	[91]
	3.12 ^c^	0 ^c^	n.a.	n.a.	6.54 ^c^	n.a.	2.74 ^c^	0 ^c^	n.a.	n.a.	2	[108]
	13.1 ^a^	37.4 ^a^	n.a.	n.a.	n.a.	n.a.	n.a.	n.a.	n.a.	n.a.	6	[109]
	25 ^k^	13 ^k^	n.a.	n.a.	n.a.	n.a.	21 ^k^	2.2 ^k^	35 ^k^	n.a.	2	[134]
	0.57 ^h^	0.16 ^h^	n.a.	n.a.	0.23 ^h^	n.a.	0.08	0.92 ^h^	0.02 ^h^	0 ^h^	2	[138]
	5.5 ^l^	1.9 ^l^	2.1 ^l^	2.2 ^l^	n.a.	n.a.	n.a.	n.a.	n.a.	n.a.	7	[141]
	0.61 ^h^	0.072 ^h^	n.a.	n.a.	0 ^h^	n.a.	0.015 ^h^	0 ^h^	0.019 ^h^	0 ^h^	9	[143]
Negroamaro (ITA)	118.2 ^a^	27.8 ^a^	n.a.	n.a.	n.a.	n.a.	n.a.	n.a.	n.a.	n.a.	1	[77]
Palomino Negro (SPA)	2.7 ^c^	0.1 ^c^	n.a.	n.a.	n.a.	n.a.	n.a.	n.a.	n.a.	n.a.	2	[122]
Pedro Ximenez (SPA)	22.4 ^f^	8.95 ^f^	n.a.	n.a.	n.a.	1.13 ^f^	n.a.	n.a.	n.a.	n.a.	12	[140]
Pinot Noir (FRA)	0 ^n^	0 ^n^	2.91 ^n^	2.42 ^n^	n.a.	0 ^n^	n.a.	n.a.	n.a.	n.a.	3	[84]
	355 ^d^	7.2 ^d^	n.a.	n.a.	n.a.	n.a.	633 ^d^	n.a.	n.a.	n.a.	10	[94]
	0 ^a^	0 ^a^	n.a.	n.a.	n.a.	n.a.	n.a.	n.a.	n.a.	n.a.	6	[109]
	2.4 ^l^	0.7 ^l^	n.a. ^*^	n.a.	n.a.	n.a.	n.a.	n.a.	n.a.	n.a.	7	[141]
Pinot Gris (FRA)	0 ^n^	0 ^n^	3.09 ^n^	2.09 ^n^	n.a.	2.07 ^n^	n.a.	n.a.	n.a.	n.a.	3	[78]
Plavina (CRO)	2.48 ^c^	0 ^c^	n.a.	n.a.	0.7 ^c^	n.a.	1.81 ^c^	0 ^c^	n.a.	n.a.	2	[108]
Primitivo (CRO)	307.1 ^a^	49.8 ^a^	n.a.	n.a.	n.a.	n.a.	n.a.	n.a.	n.a.	n.a.	1	[77]
	0 ^a^	0 ^a^	n.a.	n.a.	n.a.	n.a.	n.a.	n.a.	n.a.	n.a.	6	[109]
Sangiovese (ITA)	3.93 ^n^	0 ^n^	3.08 ^n^	2.03 ^n^	n.a.	0 ^n^	n.a.	n.a.	n.a.	n.a.	3	[84]
Syrah (FRA)	5.42 ^n^	3.02 ^n^	0 ^n^	1.9 ^n^	n.a.	0 ^n^	n.a.	n.a.	n.a.	n.a.	3	[84]
	28.7 ^a^	74.8 ^a^	n.a.	n.a.	25.39 ^a^	n.a.	n.a.	n.a.	n.a.	n.a.	5	[91]
	34.5 ^a^	0 ^a^	n.a. ^*^	n.a.	n.a.	n.a.	n.a.	n.a.	n.a.	n.a.	6	[109]
	8.5 ^k^	6.9 ^k^	n.a.	n.a.	n.a.	n.a.	8.4 ^k^	0.75 ^k^	16 ^k^	n.a.	2	[134]
	42.61 ^f^	28.64 ^f^	n.a.	n.a.	n.a.	1.12 ^f^	n.a.	n.a.	n.a.	n.a.	12	[140]
	6.4 ^l^	2 ^l^	n.a.	n.a.	n.a.	n.a.	n.a.	n.a.	n.a.	n.a.	7	[141]
Susumaniello (ITA)	147.1 ^a^	73.5 ^a^	n.a.	n.a.	n.a.	n.a.	n.a.	n.a.	n.a.	n.a.	1	[77]
Tempranillo (SPA)	165 ^b^	35 ^b^	n.a.	1.2 ^b^	9.5 ^b^	n.a.	14 ^b^	18 ^b^	n.a.	n.a.	12	[97]
	6.1 ^c^	1.5 ^c^	n.a. ^*^	n.a.	n.a.	n.a.	n.a.	n.a.	n.a.	n.a.	2	[122]
	22.87 ^f^	12.05 ^f^	n.a.	n.a.	n.a.	n.a.	n.a.	n.a.	n.a.	n.a.	12	[140]
	0.61 ^m^	0.079 ^m^	n.a.	n.a.	0 ^m^	n.a.	0 ^m^	0 ^m^	0 ^m^	0 ^m^	8	[142]
Tintilla de Rota (ITA)	1.5 ^c^	0.3 ^c^	n.a.	n.a.	n.a.	n.a.	n.a.	n.a.	n.a.	n.a.	2	[122]
Touriga Nacional (POR)	1.1 ^i^	tr ^i^	n.a.	n.a.	n.a.	0 ^i^	14.2 ^i^	0 ^i^	0 ^i^	0 ^i^	2	[144]
Touriga Francesca (POR)	0.5 ^i^	0.5 ^i^	n.a.	n.a.	n.a.	0 ^i^	5.9 ^i^	0.7 ^i^	n.a.	n.a.	2	[144]
Trnjak (CRO)	2.05 ^c^	0 ^c^	n.a.	n.a.	1.47 ^c^	n.a.	18.13 ^c^	2.54 ^c^	n.a.	n.a.	2	[108]
Uva di Troia (ITA)	127.6 ^a^	62.5 ^a^	n.a.	n.a.	n.a.	n.a.	n.a.	n.a.	n.a.	n.a.	1	[77]
Vranac (MNE)	0 ^h^	0.018 ^h^	n.a.	n.a.	n.a.	0 ^h^	n.a.	0.027 ^h^	n.a.	n.a.	2	[98]
	2.05 ^c^	0 ^c^	n.a.	n.a.	2.83 ^c^	n.a.	2.51 ^c^	2.74 ^c^	n.a.	n.a.	2	[108]
**Grapes–White Cultivars**												
Albarino (POR)	11.45 ^s^	0.23 ^s^	n.a.	2.09 ^s^	n.a.	0 ^s^	0 ^s^	8.65 ^s^	0 ^s^	8.04 ^s^	11	[87]
Chardonnay (FRA)	0 ^n^	2.95 ^n^	0 ^n^	2.25 ^n^	n.a.	2.51 ^n^	n.a.	n.a.	n.a.	n.a.	3	[84]
	63 ^o^	44 ^o^	n.a.	n.a.	n.a.	n.a.	n.a.	n.a.	n.a.	n.a.	2	[85]
	147.9 ^r^	87.5 ^r^	n.a.	n.a.	n.a.	n.a.	n.a.	n.a.	n.a.	n.a.	4	[78]
	23 ^k^	5.8 ^k^	n.a.	n.a.	n.a.	n.a.	37 ^k^	23 ^k^	n.a.	n.a.	2	[134]
Debit (CRO)	2.1 ^c^	2.22 ^c^	n.a.	n.a.	0.63 ^c^	n.a.	10.89 ^c^	4.27 ^c^	n.a.	n.a.	2	[108]
Istrian Malvasia (CRO)	8.5 ^g^	n.a.	n.a.	n.a.	n.a.	n.a.	n.a.	n.a.	n.a.	n.a.	7	[99]
Italian Riesling (NN)	6.6 ^n^	0 ^n^	0 ^n^	1.98 ^n^	n.a.	2.1 ^n^	n.a.	n.a.	n.a.	n.a.	3	[84]
Kujundžuša (CRO)	2.93 ^c^	2.08 ^c^	n.a.	n.a.	1.66 ^c^	n.a.	6.75 ^c^	1.95 ^c^	n.a.	n.a.	2	[108]
Maraština (CRO)	3.36 ^c^	1.42 ^c^	n.a.	n.a.	0.64 ^c^	n.a.	6.36 ^c^	2.8 ^c^	n.a.	n.a.	2	[108]
Moscatel (ITA)	16 ^k^	2.6 ^k^	n.a.	n.a.	n.a.	n.a.	23 ^k^	21 ^k^	n.a.	n.a.	2	[134]
	45.33 ^f^	9.39 ^f^	n.a.	n.a.	n.a.	1.86 ^f^	n.a.	n.a.	n.a.	n.a.	12	[140]
Palomino Fino (SPA)	5.99 ^f^	1.4 ^f^	n.a.	n.a.	n.a.	0.6 ^f^	n.a.	n.a.	n.a.	n.a.	12	[140]
Petra (SRB)	3.27 ^n^	3.56 ^n^	2.65 ^n^	0 ^n^	n.a.	2.15 ^n^	n.a.	n.a.	n.a.	n.a.	3	[84]
Prokupac (SRB)	0 ^n^	0 ^n^	0 ^n^	1.98 ^n^	n.a.	0 ^n^	n.a.	n.a.	n.a.	n.a.	3	[84]
Riesling (GER)	0 ^n^	0 ^n^	0 ^n^	2.01 ^n^	n.a.	2.13 ^n^	n.a.	n.a.	n.a.	n.a.	3	[84]
	14 ^k^	tr ^k^	n.a.	n.a.	n.a.	n.a.	29 ^k^	12 ^k^	n.a.	n.a.	2	[134]
Sauvignon Blanc (FRA)	0 ^n^	0 ^n^	0 ^n^	1.98 ^n^	n.a.	1.95 ^n^	n.a.	n.a.	n.a.	n.a.	3	[84]
	102.3 ^r^	210.7 ^r^	n.a.	n.a.	n.a.	n.a.	n.a.	n.a.	n.a.	n.a.	4	[78]
	9.5 ^k^	3.4 ^k^	n.a.	n.a.	n.a.	n.a.	25 ^k^	16 ^k^	n.a.	n.a.	2	[134]
Semillon (FRA)	214 ^b^	313 ^b^	n.a.	n.a.	n.a.	n.a.	n.a.	n.a.	n.a.	n.a.	2	[100]
	35.2 ^h^	1.6 ^h^	n.a.	n.a.	0.05 ^h^	n.a.	0.4 ^h^	0 ^h^	0.2 ^h^	0 ^h^	2	[138]
Ugni Blanc (FRA)	222 ^h^	3 ^h^	n.a.	n.a.	0.07 ^h^	n.a.	1.1 ^h^	0.2 ^h^	0.2 ^h^	0.04 ^h^	2	[138]
Viognier (FRA)	112.7 ^r^	105.1 ^r^	n.a.	n.a.	n.a.	n.a.	n.a.	n.a.	n.a.	n.a.	4	[78]

* Country of origin: FRA—France, POR—Portugal, ESP—Spain, ITA—Italy, CRO—Croatia, MNE—Montenegro, NN—unknown origin. ** Analysis method: 1—SLE, RP-HPLC, 2—SLE, HPLC; 3—SLE, SPE, UHPLC, 4—LLE, HPLC, 5—SLE, HPLC-PDA-MS/MS, 6—UAE, HPLC—MS, 7—SLE, HPLC—MS, 8—LC/ESI-MS, 9—SLE, LC-MS, HPLC, 10—SLE, UHPLC, 11—SPE, HPLC/QqTOF, 12—SPE, HPLC; *** n.a.—not analyzed. Results are expressed as follows: ^a^ mg/kg DW; ^b^ mg/kg; ^c^ mg/kg FW grape berries; ^d^ μg/g DW; ^e^ mg/kg berry, dry basis; ^f^ mg/g grape; ^g^ mg/L; ^h^ mg/g DM; ^i^ mg/g berry; ^j^ mg/100 g db; ^k^ mg/kg fresh grape; ^l^ mg/kg berry; ^m^ mg/g DW skins; ^n^ mg/kg frozen sample; o μg/100 g DM; ^p^ mg/100 g fresh grape; r μg/100 g FW.
